# Factors determining microbial colonization of liquid nitrogen storage tanks used for archiving biological samples

**DOI:** 10.1007/s00253-019-10242-1

**Published:** 2019-11-28

**Authors:** F. Bajerski, A. Bürger, B. Glasmacher, E. R. J. Keller, K. Müller, K. Mühldorfer, M. Nagel, H. Rüdel, T. Müller, J. Schenkel, J. Overmann

**Affiliations:** 1 Leibniz Institute DSMZ-German Collection of Microorganisms and Cell Cultures, Inhoffenstraße 7B, Braunschweig, Germany; 2 Helmholtz Zentrum München, German Research Center for Environmental Health, Institute of Developmental Genetics, München, Germany; 3 Institute for Multiphase Processes, Leibniz University Hannover, Hannover, Germany; 4 Leibniz Institute of Plant Genetics and Crop Plant Research (IPK), Seeland, OT Gatersleben Germany; 5 Leibniz Institute for Zoo and Wildlife Research (Leibniz-IZW), Berlin, Germany; 6 Fraunhofer Institute for Molecular Biology and Applied Ecology IME, Schmallenberg, Germany; 7 BioKryo GmbH, Sulzbach, Germany; 8 German Cancer Research Centre, Heidelberg, Germany; 9 Institute of Physiology and Pathophysiology, University of Heidelberg, Heidelberg, Germany; 10 Microbiology, Braunschweig University of Technology, Braunschweig, Germany

**Keywords:** Biobanking, Microbial contamination, Cryobank, Cryopreservation, Risk/quality management, Safe storage, Amplicon sequencing

## Abstract

The availability of bioresources is a precondition for life science research, medical applications, and diagnostics, but requires a dedicated quality management to guarantee reliable and safe storage. Anecdotal reports of bacterial isolates and sample contamination indicate that organisms may persist in liquid nitrogen (LN) storage tanks. To evaluate the safety status of cryocollections, we systematically screened organisms in the LN phase and in ice layers covering inner surfaces of storage tanks maintained in different biobanking facilities. We applied a culture-independent approach combining cell detection by epifluorescence microscopy with the amplification of group-specific marker genes and high-throughput sequencing of bacterial ribosomal genes. In the LN phase, neither cells nor bacterial 16S rRNA gene copy numbers were detectable (detection limit, 10^2^ cells per ml, 10^3^ gene copies per ml). In several cases, small numbers of bacteria of up to 10^4^ cells per ml and up to 10^6^ gene copies per ml, as well as *Mycoplasma*, or fungi were detected in the ice phase formed underneath the lids or accumulated at the bottom. The bacteria most likely originated from the stored materials themselves (*Elizabethingia*, *Janthibacterium*), the technical environment (*Pseudomonas*, *Acinetobacter*, *Methylobacterium*), or the human microbiome (*Bacteroides*, *Streptococcus*, *Staphylococcus*). In single cases, bacteria, *Mycoplasma*, fungi, and human cells were detected in the debris at the bottom of the storage tanks. In conclusion, the limited microbial load of the ice phase and in the debris of storage tanks can be effectively avoided by minimizing ice formation and by employing hermetically sealed sample containers.

**Electronic supplementary material:**

The online version of this article (10.1007/s00253-019-10242-1) contains supplementary material, which is available to authorized users.

## Introduction

The long-term storage of biomaterials (biobanking) is a precondition for modern life sciences, enabling follow-up scientific investigations, medical diagnostics, biotechnological applications, and the conservation of genetic resources and diversity (Overmann 2015; Overmann and Smith 2017; Schüngel et al. 2014; Stock et al. 2018). To guarantee the safe storage of biological material, dedicated quality management procedures and controls need to be improved continuously (Chatterjee et al. 2017; Lauterboeck et al. 2016; Rittinghaus and Glasmacher 2018).

Cryopreservation constitutes a key component of contemporary biobanking. Specific cryopreservation protocols have been established for different organisms and cell types. Living biological material may be prepared for cryopreservation under both, sterile or unsterile conditions. As a result, the biological materials themselves as well as the storage facilities may contain additional, accompanying organisms. For instance, plant material and human or animal cell material may be colonized by viral or bacterial pathogens (Bielanski et al. 2003; Knierim et al. 2017; Uphoff et al. 2015). Some cryopreservation techniques also require the direct contact of biomaterials with the liquid nitrogen (Rall and Fahy 1985) and are therefore particularly prone to contamination (Bielanski and Vajta 2009). However, LN and other liquefied gases are commonly manufactured in so-called air separation units which separate the atmospheric gases at very low temperatures. During this process, the air is filtered and dried. Tests conducted by one manufacturer of liquefied gases using validated methods could not detect any pathogens (personal communication Dr. Carsten Pilger, AIR LIQUIDE Medical GmbH).

So far, only anecdotal reports exist on the types of organisms occurring in LN storage tanks outside of the stored sample material. Some bacteria and fungi were determined in the debris at the bottom of LN storage tanks (Bielanski et al. 2000), but only *Stenotrophomonas maltophilia* was found also as contaminant in the cryopreserved material (Bielanski et al. 2003). An exchange of biological materials between individual samples may occur if stored in non-hermetically sealed containers in the same LN storage tank as indicated by reports of the transmission of human hepatitis B virus during cryopreservation of bone marrow transplants (Tedder et al. 1995), and by the infection of bovine embryos with bovine viral diarrhea virus and bovine herpes virus-1 after contact with contaminated LN (Bielanski et al. 2000). In a few studies, a few single microbial species were isolated directly from the LN storage tanks using culture-dependent approaches (Fountain et al. 1997; Ramin et al. 2014). However, these culture-based methods provide only very limited insights into the presence of microorganisms in complex samples since the majority of microorganisms still escapes cultivation (Overmann 2013; Overmann et al. 2017).

In the present study, we assessed the occurrence of microorganisms in LN storage tanks by state-of-the-art microscopic and culture-independent molecular approaches. In order to elucidate the types of organisms occurring in LN storage tanks, to infer possible routes of entry, and to deduce suitable strategies for quality management, we systematically screened bacteria, fungi, plant, and human cells in different phases of LN storage tanks maintained in ten different biobank facilities.

## Material and methods

### Biobanking facilities and sampling methodology

A total number of 121 samples were obtained across ten different biobank facilities in 2015 (Table S1). The LN storage tanks were located in buildings with or without air conditioning for supply and exhaust; five institutes (A, D, F, G, I) used a filtered air supply. Individual LN storage tanks varied with respect to manufacturer and type. The longest time of continuous usage without intermittent cleaning of LN storage tanks amounted to 30 years; the shortest usage interval was less than one year. Most of the tanks had not been cleaned on a regular basis in order to avoid potential damage of the stored biological materials during the transfer to another LN storage tank. Furthermore, most of the tanks had been opened regularly at least twice a week. The biological samples stored were of human (blood, stem cells), animal (rodents, fish, mussel, dove, monkey, pig, cat), or plant origin, or were microorganisms (bacteria, fungi, archaea, bacteriophages). Biomaterials were stored in cryotubes, cryobags, or straws and either in the gaseous or the LN phase of the LN storage tanks or in both (Table S1).

**Fig. 1:**
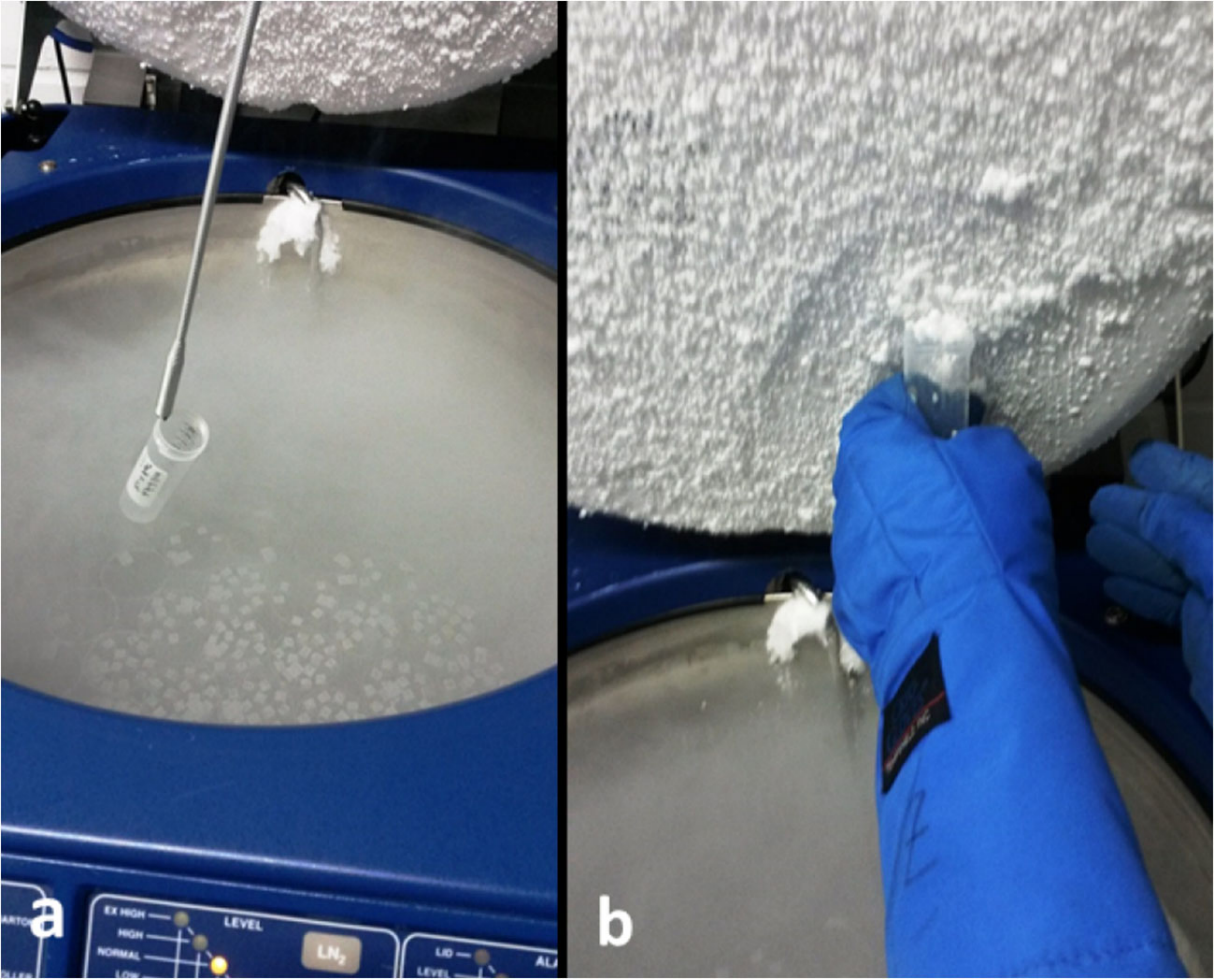
Liquid nitrogen (LN) storage tanks and sampling procedure of LN and ice. (**a**) Sampling of the LN phase using a reaction tube and grip tongs. (**b**) Sampling of the ice phase formed underneath the lids (and rim)

Wherever accessible, the LN phase, ice layers underneath LN storage tank lids, and debris accumulated at the bottom of LN storage tanks were sampled (Figs. 1a, b). For each LN sample, 15 individual subsamples, each amounting to 50 ml LN, were collected in Falcon tubes (Fig. 1a). The LN subsamples were incubated until all LN had evaporated. Ice samples were scraped off the inner rim or from the bottom face of the lid into a Falcon tube (Fig. 1b). Each ice sample amounted to 10–100 ml of thawed ice depending on accessibility. LN and ice samples were collected in three consecutive months (Table S1). All samples were stored frozen and shipped on (dry) ice to the Leibniz Institute DSMZ for subsequent analyses.

For further processing, of each LN sample, residuals from eleven pooled 50 ml-subsamples (total 550 ml) were used for DNA extraction. A total volume of 10 ml of 4-(2-hydroxyethyl)-1-piperazineethanesulfonic acid (HEPES; Serva, Heidelberg, Germany) buffer (25 mM, pH 7.3) was added and the tubes were incubated at room temperature on a horizontal shaker to resuspend the residuals for 15 min. Samples were filtered through a 0.1 μm pore-size polycarbonate filter and stored at −20 °C for DNA extraction. For microscopic counting, the residuals from four pooled 50 ml (total 200 ml) LN subsamples were resuspended in 5 ml HEPES by shaking for 15 min and then fixed with glutaraldehyde (final concentration, 2% w/v; Serva, Heidelberg, Germany). The ice samples were thawed and HEPES buffer was added up to 10 ml in samples containing less than 10 ml thawed ice. An aliquot of 1.8 ml of each sample was fixed with glutaraldehyde for microscopic counting and the remaining suspension filtered through a 0.1 μm pore-size polycarbonate filter.

Three types of negative controls were included. Firstly, empty Falcon tubes provided along with the samples by each participant served as negative controls for the contamination of laboratory equipment, they were filled with 25 ml HEPES (Negative Control = NC eq). Secondly, reference samples processed at DSMZ consisting of 550 ml of the LN were filled into a sterilized Dewar, 0.1 μm Isopore™ polycarbonate filters (Merck Millipore Ltd., Tullagreen, Carrigtwohill, Irland) added, and the LN evaporated (NC ref). Thirdly, two of the 0.1 μm polycarbonate filter were treated by filtering 25 ml HEPES (NC HEPES). All controls were processed in parallel and in the same way as the samples.

### Microscopy

For fluorescence microscopy, fixed cells were collected onto polycarbonate filters (25 mm diameter; 0.1 μm pore size), the filters were stained with 50 μl SYBR Green I (LifeTechnologies, 1:10000 in DMSO; Sigma-Aldrich, Darmstadt, Germany) and mounted in a drop of immersion oil on a glass slide. The samples were analyzed using a Zeiss (Oberkochen, Germany) Axio Imager.M2 microscope at excitation/emission wavelengths of 470/525 nm, and the Axio vision software Rel. 4.8.2. Twenty microscopic fields were counted in triplicate for each sample. Conspicuous structures were analyzed further for the presence of chlorophyll *a* autofluorescence as indicator of the presence of algae or plant cells using a Nikon (Düsseldorf, Germany) Ti microscope at an excitation wavelength of 425 nm and an emission wavelength of 607 nm and Nikon software NIS-Elements AR 4.13.01.

### DNA extraction and PCR

DNA was extracted from the filters using the DNA Micro Kit (Qiagen, Hilden Germany) according to the protocol of the manufacturer. Filters were cut into strips and incubated with lysozyme (final concentration, 20 mg per ml; Serva, Heidelberg, Germany) at 37 °C on a shaker (800 rpm) for 1 h. In the second lysis step, 20 μl proteinase K (final concentration 50 μg per μl; Applichem, Darmstadt Germany) was added and the samples were incubated at 56 °C over night. In the final step of the protocol, DNA was eluted in 20 μl PCR-clean water (Promega, Mannheim, Germany).

Bacterial 16S rRNA genes, eukaryotic (human) transposable elements Line1, and fungal ITS region were PCR amplified using the respective primer sets 8F-1492R, Line1 and ITS1F-ITS4 at a final concentration of 0.2 pmol per μl (Table S2). The PCR was performed in an Applied Biosystems cycler (Foster City, USA) using Thermo Scientific DreamTaq Green (0.02 U per μl; Waltham, USA) and buffer (Table S2). Bacterial 16S rRNA gene copy numbers (V3 region; specific primers at 0.2 pmol per μl final concentration, Table S2) were determined in a quantitative real-time PCR using LightCycler® (Roche, Basel, Schweiz) 480 and SYBR Green I. *Mycoplasma* was detected using a previously established PCR-based detection method (Uphoff and Drexler 2002). This endpoint PCR was performed in an Applied Biosystems cycler using Invitrogen Platinum Taq (0.02 U per μl; Carlsbad, USA) and buffer (Table S2).

### **Library preparation and sequencin**g

The V3-region of the bacterial 16S rRNA gene was sequenced using amplicons generated with specific primers 341F wobble and 515R (0.2 pmol per μl each), Qiagen Phusion polymerase (0.04 U per μl; Hilden Germany) and GC-buffer with the addition of dNTPs (0.2 mM), BSA (0.8 mg per ml), MgCl_2_ (0.5 mM), DMSO (3.0%), and PCR-clean water. Between 1 and 20 ng, DNA template was used. The PCR product (60 μl) was cleaned up using DNA Clean & Concentrator™-5 (ZymoResearch, Irvine, USA) eluting the product in 30 μl water. After adding 0.1X TE (1 mM Tris-HCl, pH 8.0, 0.1 mM EDTA) to a final volume of 50 μl, the amplicon was processed using the NEBNext® Ultra™ II DNA Library Prep Kit for Illumina® (New England Biolabs, Frankfurt a. Main, Germany) according to the protocol of the manufacturer. Amplicons were prepared for adapter ligation using the NEBNext End Prep enzyme mix, and the 25-fold diluted adapter was ligated in a subsequent step. Adapter-ligated fragments were cleaned up without size selection using Agencourt AMPure XP Beads (Beckman Coulter GmbH, Krefeld, Germany). Then, the adapter-ligated DNA was enriched by 13 PCR cycles of using NEBNext® Multiplex Oligos for Illumina® (Index Primers Set 1, New England Biolabs, Frankfurt a. Main, Germany). The size distribution of the purified PCR product (AMPure XP Beads) was checked on an Agilent Bioanalyzer (high sensitivity chip; Santa Clara, USA). Adapter dimers of the combined library pool (~ 10 ng PCR product per sample) were removed by gel purification (MetaPhor® agarose; Lonza, Basel, Switzerland) using the NucleoSpin® Gel and PCR Clean-up Kit (Macherey-Nagel, Merck, Darmstadt, Germany) and amplification products were sequenced on a HiSeq 2500 Ultra-High-Throughput Sequencing System (Illumina, San Diego, CA, USA) as described recently (Gossner et al. 2016).

Raw sequence reads were organized based on unique barcodes and denoised into amplicon sequence variants (“sequence variants” in the following) using plugins implemented in Quantitative Insights into Microbial Ecology (Qiime2, ver. 2017.12.0; Caporaso et al. 2010; team 2016-2018 https://qiime2.org/) creating a Feature Table. Default settings were used unless otherwise noted. The forward and reverse reads were joined, chimera-filtered and clustered (vsearch, Rognes et al. 2016), quality filtered (Bokulich et al. 2012) and trimmed to a length of 150 bp (minimum size = 2, minimum reads = 5; deblur, Amir et al. 2017). A phylogenetic tree was constructed with FastTree (Price et al. 2010) after performing multiple sequence alignment using MAFFT (Katoh and Standley 2013) and Mask (Bailey and Gribskov 1998). Samples were rarefied to 99.0% sequence coverage (Chao and Jost 2012). Taxonomy was determined using a pre-trained Naive Bayes classifier based on the SILVA Database (v.128, Quast et al. 2013; Yilmaz et al. 2013) with the Qiime 2 plugin feature-classifier (https://github.com/qiime2/q2-feature-classifier). The reads were then compared against SILVA 132 SSURef Nr99 with an initial identity cutoff of 97% with vsearch 2.7 (-strand both, Rognes et al. 2016). Each read was then taxonomically assigned to the hit with the best bit score. When multiple best hits were present, the first one listed was chosen. The origin of the sequence variants was analyzed using the *microbial isolation sources search* implemented in bacterial metadatabase Bac*Dive* (Reimer et al. 2018). All Illumina datasets were submitted to the SRA database under accession number PRJNA558333.

### Statistics

Statistical tests were performed using R (version 3.3.4, R-Core-Team 2017). Two-sample t-test and variance F-test were calculated for the gene copy numbers and relative abundances of single taxa comparing reference and single LN storage tank samples. The variance between different groups was determined by one-way-ANOVA with multiple comparisons of means using Tukey Contrasts (package multcomp, Herberich et al. 2010) shown as compact letter display (cld, Piepho 2004). Correlations for the association between paired samples were tested (R, corr.test) using two-sided Spearman's rank correlation rho. A multivariate analysis of variance of the distance matrices was performed with permutation tests (*n* = 999) using the adonis2 function of the vegan package in R (McArdle and Anderson 2001). Different linear models (generalized with mixed effects, Kuznetsova et al. 2015) were applied to evaluate the effect of predictor variables (institute, storage phase, surrounding condition, stored material, storage device, number of openings and usage time) on a response variable (gene copies or cell numbers). The Akaike information criterion (AIC) and residual plots (DHARMa, Hartig 2017) were taken into account. All response variables were log-transformed.

Employing the phyloseq package (McMurdie and Holmes 2013) of R (version 3.3.4, R-Core-Team 2017), a principal coordinates analysis (PCoA) based on weighted UniFrac distances was calculated on species level for sequence variants defined at 3% sequence dissimilarity. Student's t-Test was performed to compare the weighted UniFrac distances between samples above (ice, debris) and below the threshold of the negative controls (NC, LN). For further analysis, the values determined for negative controls were taken as a threshold. Therefore, all samples containing cell counts of < 10^2^ cells per ml were excluded from the analysis. Alpha-diversity (Chao1, Shannon diversity index; for institute comparison all samples and NCs were included), Constrained Analysis of Principal Coordinates (CAP, based on weighted UniFrac distances), and relative abundances of the bacterial communities were calculated on genus level removing sequence variants with abundances of less than 5 reads per sample phyloseq package (McMurdie and Holmes 2013) of R (version 3.3.4, R-Core-Team 2017). The parameter cells per ml, surrounding condition (building), storage phase, time of usage, frequency of openings, storage device, and stored material were used as constraining variables.

## Results

### Microbial cell counts and PCR detection

**Fig. 2:**
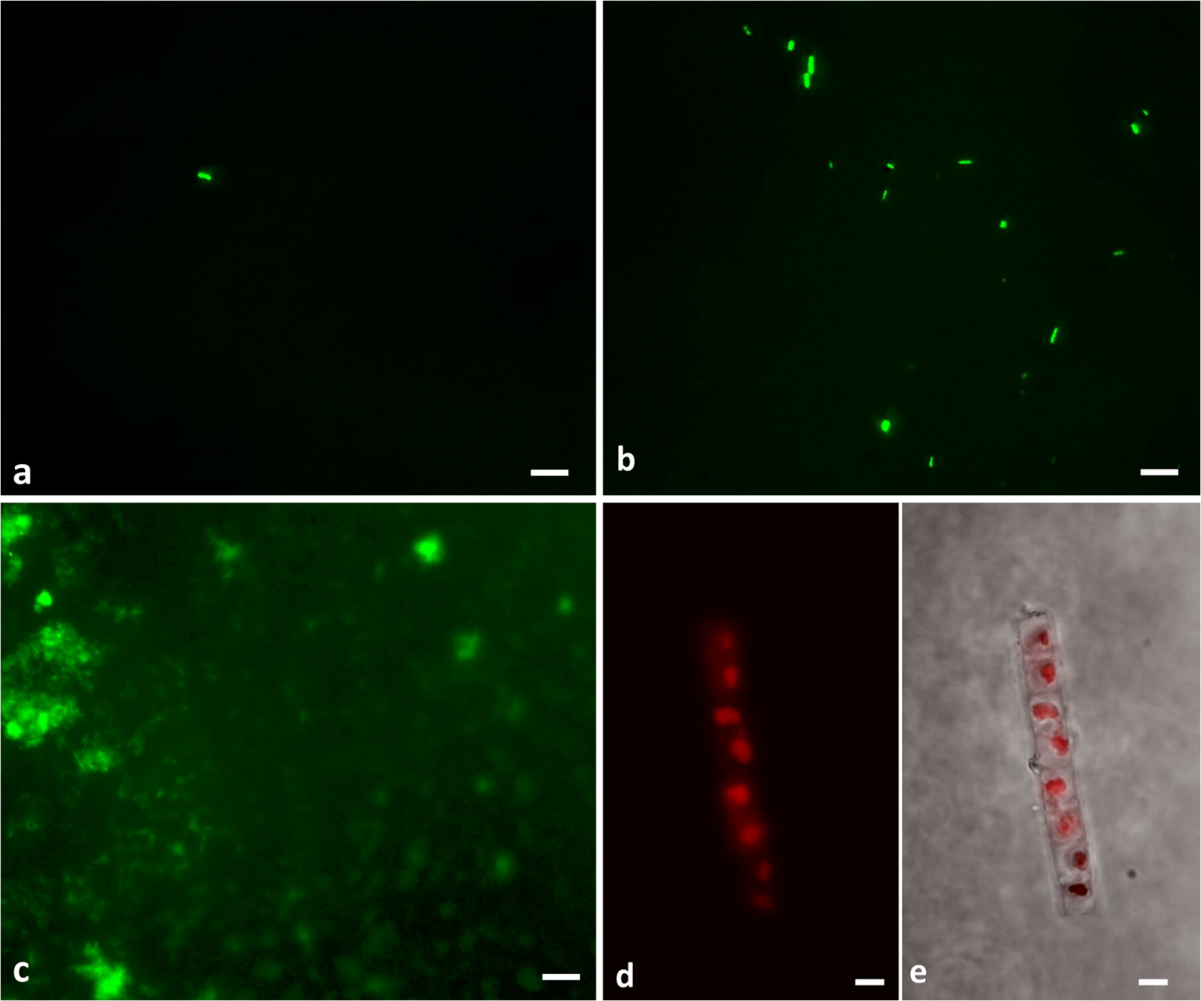
Epifluorescence photomicrographs of SYBR Green I-stained samples and algae autoflourenscence. (**a**) bacterial cells detected in the negative control NC-B-2 containing 2 × 10^2^ cells per ml, (**b**) bacterial cells detected in the ice sample B-4-2 containing 8 × 10^3^ cells per ml, (**c**) eukaryotic cells of sample G-20-1, confirmed by PCR with Line1 primer, filaments of *Cyanobacteria* in sample I-30-3 (**d**) autofluorescence of microbial cells (excitation 425 nm, emission 630 nm) and (**e**) overlay with phase contrast. Sample code, institute-identity number-replicate; Scale bar, 5 μm

**Fig. 3:**
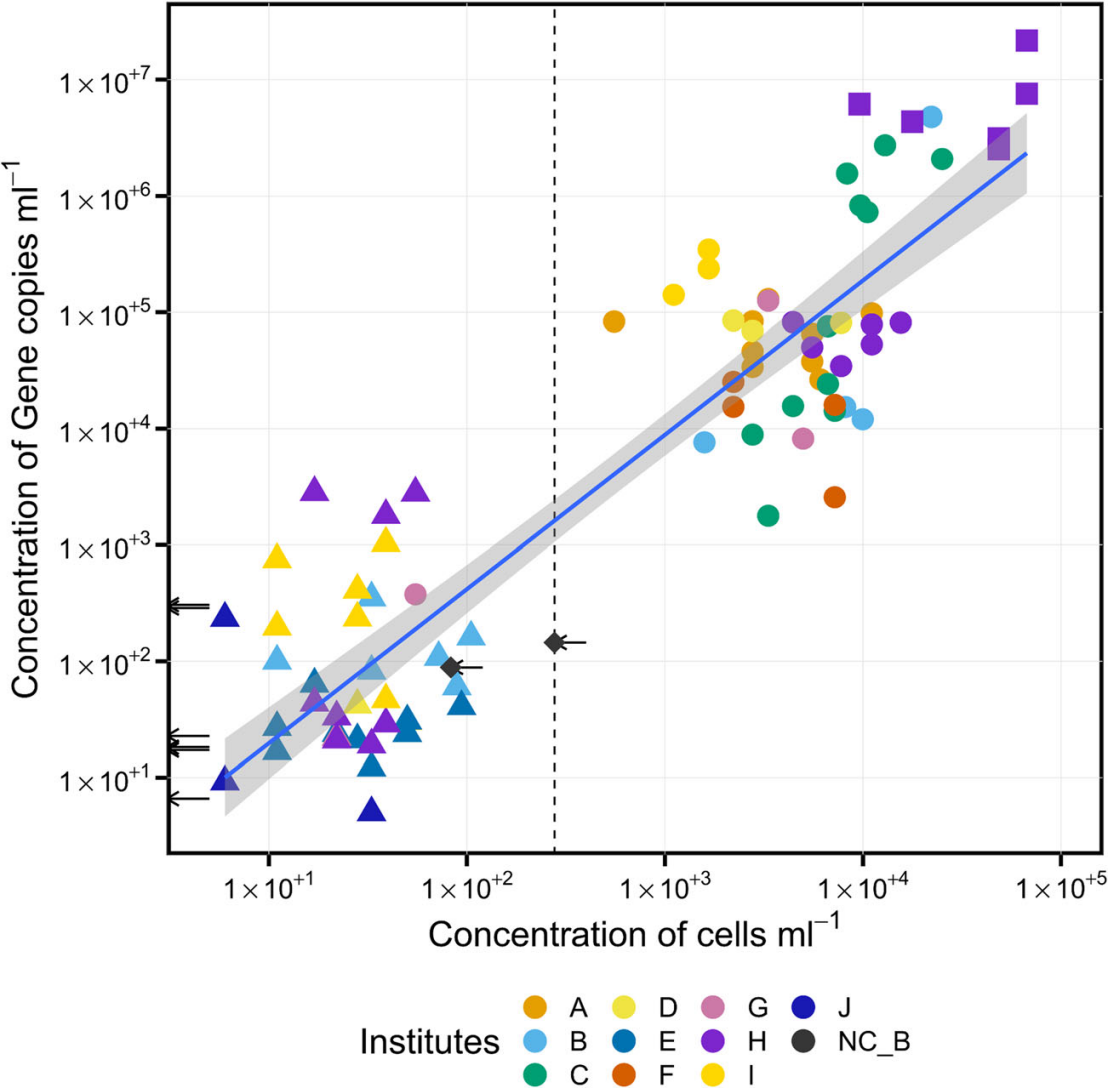
Correlation of gene copy numbers and cell counts. A clear separation of ice (circles) and LN (triangles) samples could be observed. The arrows indicate the gene copy numbers of the negative controls. A clear separation of ice (circles) and LN (triangles) samples could be observed. The negative control (diamonds, NC_B) with the highest cell number determined the threshold for the detection limit, illustrated by a vertical dashed line. The axes are log_10_-scaled. The LN samples are in the range of the negative controls. The debris samples (squares) had the highest gene copy numbers and cell counts. The concentration is calculated per ml evaporated LN, thawed ice, air volume reaction tube (NC_B)

Bacterial cell counts in both, negative controls as well as in the LN samples were low and at ≤ 10^2^ cells per ml LN (Figure 2a, Table S3). Correspondingly, all LN samples and negative controls showed 16S rRNA gene copy numbers of ≤ 10^3^ per ml LN (Table S3, Fig. 3). Variance analysis of gene copy numbers between the different sample types showed that samples taken directly from the LN phase could not be distinguished from the negative controls (*p* = 0.158, ANOVA, Table S4), indicating that the microbial entry in the LN samples is below the detection limit determined by the negative controls and reference samples. The negative control (NC_B) with the highest cell number determined the threshold for the detection limit (Fig. 3). Therefore, a threshold of 277 cells per ml LN was applied to choose samples to be included in all subsequent analyses. All samples which had cell numbers below the threshold were only considered for selected comparative analyses, in particular to access the potential origin of the cells.

In contrast to the LN phase, cell numbers in ice or in debris samples were up to 100 times higher (up to 10^4^ cells per ml ice, Fig. 2b, Fig. 3, Table S3). The ice samples contained between 10^3^ and 10^6^ 16S rRNA gene copies per ml ice and thus significantly surpassed the values of LN samples (*p* = 0.019) and of the negative controls (*p* = 0.019, Table S4). The calculated ratio of cell concentration to 16S rRNA gene copies per ml was between 1 and 10 (Fig. 3).

The concentration of cells and gene copies clearly showed an institute-related pattern indicating an influence of the characteristic parameters of each tank or institute (Fig. 3). In a generalized linear model using Gaussian distribution, the institute, storage phase, surrounding condition, number of openings, and the usage time predicted the presence of cells (AIC = 96.3, KS test: *p* = 0.1666) and gene copies (AIC = 168, KS test: *p* = 0.5457, Table S5). The two variables “storage material” and “storage device” were included in the model as well, but their effect was redundant with the variables listed above. The observed institute pattern (Fig. 3) was supported by the linear model (Table S5) which identified the institutes as main predictor variables. Specifically, the response variables “cell number” and “gene copy numbers” were higher in institute A (cells), B (copies), C, D, F, and H but were lower in institute I (Table S5, Fig. S1a). Additionally, cells and gene copies increased with storage time (Fig. S1d) and number of openings (Fig. S1c). The numbers of bacteria were lower in rooms with air supply and exhaust but higher in the debris samples (Fig. S1b) and in tanks, where the material is stored in the LN phase (Table S5, Fig. S1).

Of all samples yielding bacterial PCR products, over 20% were also tested positive for 16S rRNA genes of *Mycoplasma*. However, *Mycoplasma-*DNA was only detected at very low abundances, accounting for up 1–3% of the 16S rRNA gene copies, freely occurring *Mycoplasma* cells were not detected in this study.

Fungal ITS sequences were present in 19 ice and debris samples from 5 institutes (Table S3 and Table S6) and predominately occurred in tanks containing mixed materials stored (institutes F, H, I), as well as when stored in the gaseous nitrogen phase (institutes C, I) and even in tanks located in rooms supplied with filtered air (institutes F, I). In several samples, a few eukaryotic cells could be detected by SYBR Green I staining (institute-identity number: A 2, A 3, B 5, B 7, C 11, F 18, G 19, G 20, I 28, I 29,and I 30 (Fig. 2c, J 31). Accordingly, human cells were detected in samples B 5, B 7, C 11, F 18, G 20, I 29, and I 30 by specific PCR with Line1 primers (institute-identity number, Table S3). *Mycoplasma*, fungi, and human cells were not detectable by the specific PCR protocols in any of the negative controls.

Plants or algae were not targeted by a specific PCR protocol since chloroplast sequences were already covered by bacterial 16S rRNA gene sequencing. Using this approach, chloroplast sequences (affiliated with the phylum cyanobacteria) could be detected in over 90% of the samples from all institutes (Fig. S2) with exception of the debris samples from institute H. While chloroplast sequences occurred only in traces (< 1% of the total abundance) in most (70%) of the samples, about 25% of the samples contained more than 10% chloroplast sequences in their sequence dataset. Among those were all samples of institute I where a mixed set of materials (plants leaves, fish, mussels, dove eggs) are stored. Accordingly, algae cells were observed by epifluorescence microscopy in samples from tanks of institute I (Fig. 2d-e and Fig. S3). Overall, the presence of cyanobacteria/chloroplast sequences was related to the stored material. They occurred predominately in tanks that harbored plant material, either exclusively or next to other eukaryotes, but also in tanks storing animal material (Fig. S4).

### Diversity and taxonomic composition of bacterial communities

The bacterial species richness determined on the genus level for individual LN storage tank samples typically stayed below 300 sequence variants. Higher values were only determined in five individual samples (Fig. S5a). Using Chao 1 as an estimator of total species richness and considering the background of species that is introduced through consumables and/or chemicals (50–70 sequence variants), less than 200 sequence variants of bacteria typically had accumulated over time in the majority of the LN storage tanks (Figure S5b). Overall, the α-diversity of samples (all sample types combined) from institutes A, F, and G was significantly different from the other samples and the negative controls since they showed an average number of about 200 sequence variants and a Shannon diversity index (Fig. S5c, Table S7) between 3 and 3.5, whereas the number of sequence variants in samples from institutes D and E matched the determined background of species. The LN storage tanks of institutes H and I showed a high variability in α-diversity, ranging from 50–350 sequence variants and a Shannon index of 2.0 to 4.5 in samples from institute I, and 1–200 sequence variants and a Shannon index of 0.1 to 4.0 in samples from institute H. Excluding all samples with bacterial counts below the determined background, the three variables “opening frequency,” “sampled phase” (LN, ice, debris), and “stored material” were identified as main determinants of species richness and diversity (Table S7). Thus the number of sequence variants (and the diversity) decreased from 300 in tanks opened daily to less than 50 in tanks opened once a month, but the intragroup variability was still high. Tanks opened more seldom did not follow the trend (data not shown). The opening frequency was positively correlated to α-diversity (*p* < 0.01, excluding seldomly opened tanks).

Notably, species richness and diversity were low in the debris samples (< 50 species) despite the relatively high cell numbers in those samples. Samples storing mixed material showed significantly higher species richness and diversity than samples storing animal material (Table S7). The environmental conditions and the type of storage phase only had a minor impact on the observed number of sequence variants and no effect on Shannon diversity (Table S7). Only LN storage tanks mounted in a hall had significantly higher species richness.

**Fig. 4:**
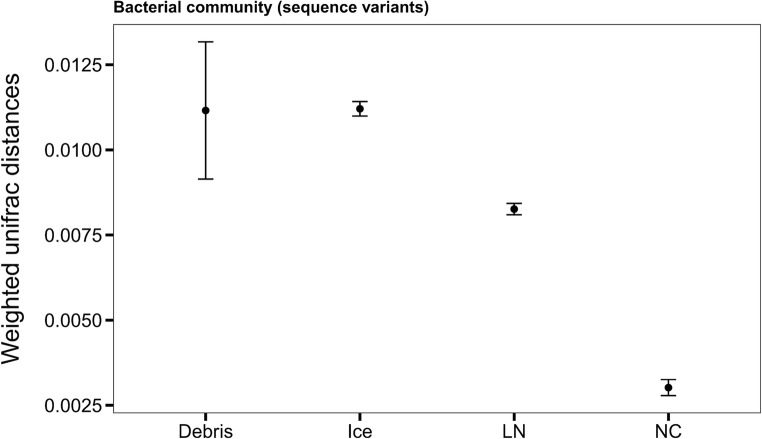
Weighted UniFrac distances of bacterial sequence variants at species level. Shown are the distances grouped by the samples phase: debris, ice, liquid nitrogen (LN) and the negative controls (NC)

Upon analysis of the bacterial community structure with PcOA, about 50% of the species distribution could be explained by the first two axes (Fig. S6). The variables “institute”, “sampled phase”, “storage phase”, “condition”, “stored material”, “storage device”, “opening”, and “usage time” shaped the bacterial community significantly (permutations = 999, *p* < 0.001). Strengthening these results, the three consecutive samplings of the same LN storage tank did not yield significant differences in the bacterial community composition. Bacterial communities detected in the ice phase and in debris were more variable than those detected in the LN phase or the negative controls (t-test, *p* < 0.001, Fig. 4). The LN phase and the negative controls had a highly similar composition, supporting once more a common origin of these 16S rRNA gene sequences from the same source, most likely plastic ware consumables or chemicals. Therefore, the determinants of the bacterial community composition in the ice layers and debris of LN storage tanks were analyzed in further detail.

**Fig. 5:**
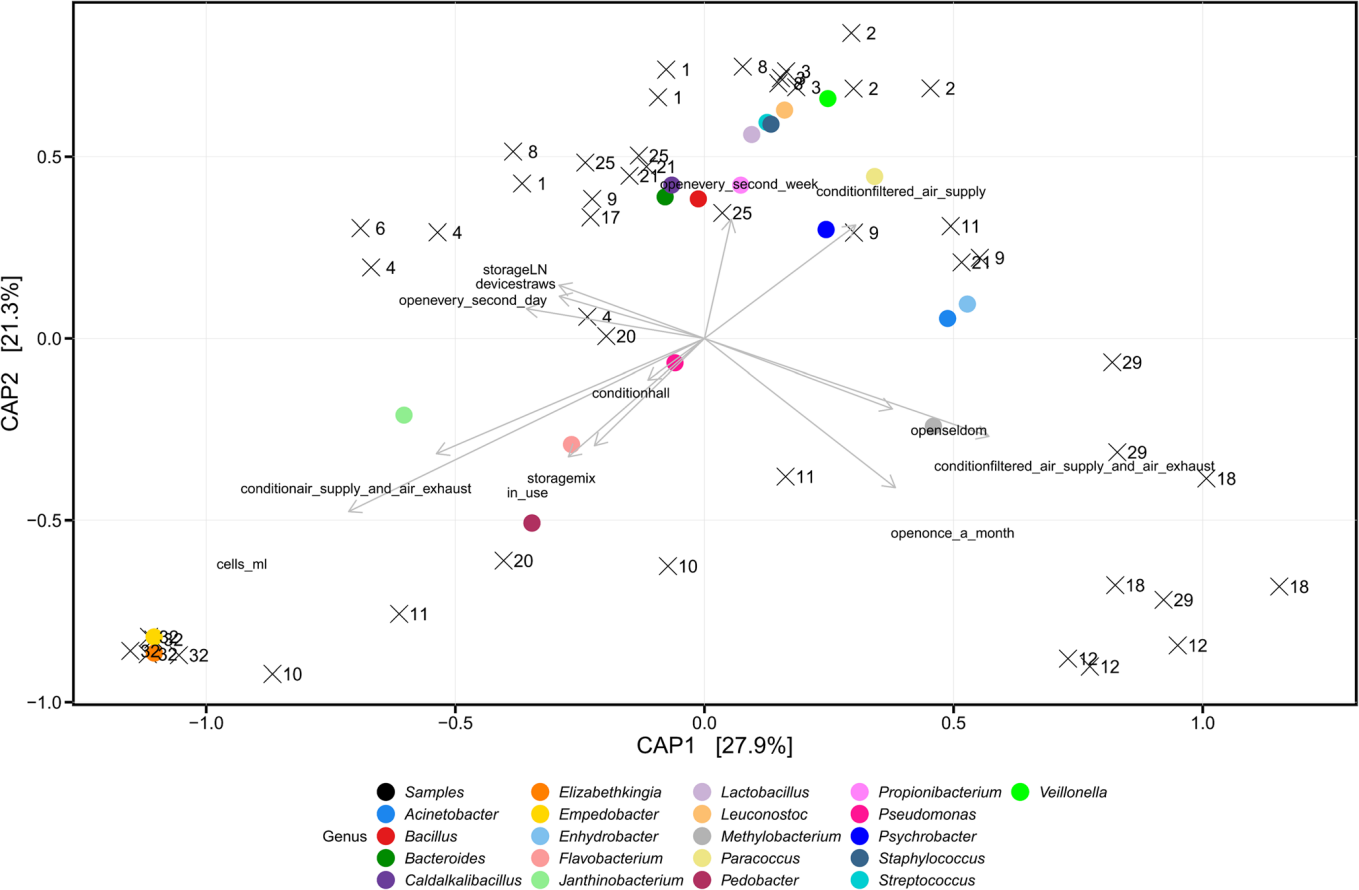
Bacterial community structure shaped by environmental parameters determined by a constrained analysis of principal coordinates (CAP) of a selected data set. The NC samples and all samples with < 277 cells ml^−1^ were excluded from the analysis. OTUs that do not appear more than 5 times in at least 1% of the samples were removed. The CAP was calculated based on weighted UniFrac distances. The parameter cells_ml x + condition + storage + copies + in_use + open + storage_device + material were used as constrained variables. Black colored shapes depict samples encoding for different institutes including the ID shown as numbers. Colored circles depict the selected taxa encoding for the 20 most abundant genera

For this purpose, the ordination was constrained by the environmental variables. About 48% of the variability was explained by the first two principle coordinates (Fig. 5, permutations = 999, *p* < 0.03) and the variability in the 20 most abundant genera could be assigned to certain conditions and samples. The opening frequency seemed to influence the bacterial community of LN storage tanks in institute C, separating LN tanks No. 10 and 11 from No. 8 and 9. Interestingly, samples that were tested positive for fungi and *Mycoplasma*-DNA with PCR also harbored a specific bacterial community or particular bacterial taxa.

The most abundant genera were *Methylobacterium*, *Bacteroides*, and *Caldial kalibacillus*. Sequences affiliated with *Methylobacterium* were detected in many samples and reached a relative abundance of up to 80% in samples 12 and 29 (Fig. S7, Table S6). *Methylobacterium komagatae* was present in several LN storage tanks (e.g., samples No. 12, 36), while in sample No. 18 that clustered in the same group of bacterial communities as samples No. 12 and 36 (Fig. 5), *Methylobacterium populi* sequences were predominant. Strains such as *Ralstonia pickettii* (*Betaproteobacteria*), *Bradyrhizobium* (*Alphaproteobacteria*), *Pseudomonas saccharophila* (*Gammaproteobacteria*), *Sphingomonas* (*Alphaproteobacteria*), and *Bacillus* (*Bacilli*) are typical representatives of the most abundant phyla in the LN storage tanks (Fig. S2). Within the genus *Streptococcus*, the most abundant sequence variants were affiliated with *Streptococcus pneumoniae* (identity No. 1, 2, 3, 8, Table S5). The distribution of *Staphylococcus* was similar to those of *Streptococcus* and the most abundant sequence variants belonged to the species *Staphylococcus equorum* (No. 8), *Staphylococcus epidermidis*, and *Staphylococcus pasteuri* (identity No. 1, 2, 3, 8, 25). Most of the sequences of sample No. 20 were affiliated to *Pedobacter glacialis* (Qiu et al. 2014), and many *Flavobacterium* spp. were observed in tanks containing mixed biomaterials (No. 20, 30, Fig. 5 and S5, Table S5). A few genera occurred at very high relative abundance but only in one individual LN storage tank. *Elizabethkingia*, *Empedobacter*, and *Janthinobacterium* were almost exclusively found in the debris sample (No. 32) from a single LN storage tank of institute H storing microorganisms (Fig. 5, Fig. S7) and where the 16S rRNA gene amplicons of all three bacterial genera reached relative abundances of 42%, 35%, and 17%, respectively (Fig. S7). Similarly, *Flavobacterium succinicans* and *Flavobacterium psychrophilum* were only detected in a LN tank in which fish samples were stored in glass flasks.

## Discussion

### Detection of microorganisms in LN tanks

Bacterial cells and 16S rRNA gene copy numbers were hardly detected in controls and LN samples, while cell numbers in ice or in debris samples significantly surpassed the values of LN samples and the determined detection limit. The detection limit attained in the present study is on the low end of that reached by cultivation-dependent methods. For example, between 10^2^ and 10^5^ colony forming units per ml melted sediment were found in different Dewars from in vitro fertilization clinics (Morris 2005). The detection limit of our qPCR approach is in the range of the detection limit of 100 copies for qPCR analysis (corresponding to 10^4^ to 10^5^ colony forming units per square meter per sample) that has been proposed for cleanrooms (La Duc et al. 2007).

The calculated ratio of cell concentration to 16S rRNA gene copies per ml corresponds to an average number of up to 10 rRNA operons per cell, which is in line with the typical values reported for bacteria (Klappenbach et al. 2000; Rainey et al. 1996). These results substantiate that the two measures for bacterial abundances employed in the current study yielded consistent results.

Our highly sensitive methods also allowed the phylogenetic assignment of bacterial DNA in the negative control, which might be introduced by laboratory equipment, chemicals, DNA kits, or sequencing equipment. In accordance with our results, it has been reported that bacterial contaminants present in commercial DNA sequencing kits may reach values of 10^3^ cells per sample, mainly from the bacterial genera *Methylobacterium*, *Pseudomonas*, *Streptococcus*, *Acinetobacter*, *Bacillus*, *Psychrobacter*, *Propionibacterium*, *Variovorax*, *Flavobacterium*, *Enhydrobacter*, *Corynebacterium*, *Janthinobacterium*, *Pedobacter*, and *Burkholderia* − *Paraburkholderia* (Salter et al. 2014) and that samples containing less bacterial cells need to be excluded to avoid interference of analysis background contaminants with bacterial community analysis.

### Occurrence patterns of different groups of organisms in the LN tanks

Accompanying bacteria play important roles for the safety status of collections. The numbers of bacteria increased with storage time and number of openings. This is in contrast to the study of Morris et al. (Morris 2005), in which the microbial load was not correlated with usage time. Fungal DNA in ice and debris samples occurred in tanks storing mixed materials, as well as when stored in the gaseous nitrogen phase and even filtered air systems did not prevent the presence of fungi (DNA) completely. These results are in line with previous studies reporting a predominance of fungi in commercial operated tanks that stored samples in the vapor phase (Bielanski et al. 2003; Fountain et al. 1997).

The genus *Mycoplasma* is a known contaminant of cell lines and therefore of particular relevance for the cryopreservation of human cell lines (Drexler and Uphoff 2002). In this study, *Mycoplasma* was only detected on the molecular level at very low abundances. Nevertheless, a *Mycoplasma* contamination needs to be avoided which can be achieved by screening of eukaryotic sample material before cryostorage (which is already done in many biobanks), especially for sensitive applications like transplants, because a *Mycoplasma* infestation renders the biological resource useless in these cases and results in a financial burden. Nevertheless, based on the results of this study, the rather rare occurrence of fungi and eukaryotic cells is expected to have little effect on biobanking. Chloroplasts occur more frequently than the other specific groups investigated, especially when mixed samples are stored. Yet the accompanying plant materials are not considered as a threat for the stored bioresources since they typically are not pathogenic. The exceptional high amount in single samples may be caused by material released due to failure of container seals.

In summary, the microbial cell concentration was clearly depending on the institutes and their specific storage conditions. We therefore conclude that individual measures taken by biobanking institutes exert a profound effect on the quality of the cryostored biomaterials.

### Distribution patterns of bacterial taxa in the LN tanks and their determinants

The bacterial species richness for LN storage tank samples is much lower compared to species richness of soils (Bach et al. 2018) or of more selective bacterial habitats such as pig carcasses (Pascual et al. 2017) that harbor around 750 or 500 sequence variants on the genus level (97% similarity), respectively. A low species richness and diversity in the debris samples despite the relatively high cell numbers indicate that the bacteria in debris were introduced by single escape event like the breakage of glass capillaries containing a single bacterial strain.

The presence and abundance of several of the bacterial taxa observed in the LN tanks can be explained by their (1) frequent occurrence in the environment, (2) presence on/in the operating personnel, (3) their specific physiological adaptations, or (4) the introduction into LN tanks in association with the stored biomaterials.

Representatives of the genus *Methylobacterium* are able to form biofilms that tolerate disinfecting agents, high temperatures, or low water availability and as a result occur widespread in man-made environment such as industrial storage tanks (Kelly et al. 2014) as well as in the clinical environment (Kovaleva et al. 2014). *Methylobacterium komagatae* has been isolated from water samples taken from food-manufacturing plants (Kato et al. 2008) and in this study was also present in LN storage tanks. A reservoir of *Methylobacterium* within the premises and cell dispersal, e.g. by air circulation, might therefore explain the dominance and distribution of this genus in the LN storage tanks studied. Most of the *Methylobacterium* sequences were affiliated with the plant-associated *Methylobacterium populi* (DOI:10.13145/bacdive133659.20180622.3). They might originate from the stored plant material, which was stored in non-hermetically sealed tubes in the respective LN tank. Devices which are not hermetically sealed may allow the exchange between the cryopreserved materials and the surroundings (Tedder et al. 1995).

*Pseudomonas*, *Bacilli*, or *Acinetobacter* occur widely distributed in nature and are common in soil, water, and plants but also in technical systems (such as *Acinetobacter johnsonii* from spacecraft-associated clean rooms; Moissl-Eichinger et al. 2012). These bacteria may therefore enter the LN storage tanks via the technical environment (air, water, filter, and supply systems) or even originate from the stored plant materials. Several of the *Pseudomonas* sequences detected are affiliated with environmental (*Pseudomonas stutzeri*) or plants-associated species (*Pseudomonas syringae*; (Buell et al. 2003)).

Strains of *Ralstonia pickettii* (*Betaproteobacteria*), *Bradyrhizobium* (*Alphaproteobacteria*), *Pseudomonas saccharophila* (*Gammaproteobacteria*), *Sphingomonas* (*Alphaproteobacteria*), and *Bacillus* (*Bacilli*), which are typical representatives of the most abundant phyla in the LN storage tanks, have also been isolated from ultrapure water in industrial systems (Kulakov et al. 2002; Mijnendonckx et al. 2013). Ice crystals in LN tanks and aerosols may capture airborne environmental bacteria and were previously suggested as a potential contamination source in culture-based studies (Bielanski et al. 2003; Morris 2005). Whereas the commercial produced LN is supposed to be pathogen-free, LN supply systems may not be sterile and could thus become a source for microorganisms in LN tanks (Bielanski and Vajta 2009).

Several of the 20 most abundant genera (Fig. S7) are typically associated with humans or animals. Thus, members of the highly abundant and frequently occurring genus *Bacteroides* are dominant members of the mammalian gastrointestinal microbiome (Ryan and Ray 2004). Indeed, several of the *Bacteroides* sequences identified were affiliated with previously described isolates from fish and fecal samples (Kabiri et al. 2013). All identified streptococcal sequence variants were human or mammal-associated and are part of the commensal microbiota of the mouth, skin, intestine, and respiratory tract as well as the salivary microbiome (Wang et al. 2016). The identified species *Staphylococcus equorum* (No. 8), *Staphylococcus epidermidis*, and *Staphylococcus pasteuri* are part of the commensal mammal microbiota of skin, hair, and nail. *Staphylococcus pasteuri* has even been isolated from clean rooms (Moissl-Eichinger et al. 2012). Similarly, *Propionibacterium* is a commensal bacterium on the human and animal skin (Stackebrandt 2014). Thus, members of the abundant bacterial genera *Bacteroides*, *Streptococcus*, *Staphylococcus*, and *Propionibacterium* in the LN tanks likely originated from operating or other personnel. Our results also confirm the results of previous investigations that used cultivation approaches and detected *Pseudomonas*, *Streptococcus*, *Acinetobacter (*e.g., *Acinetobacter calcoaceticus)*, *Bacillus*, *Propionibacterium*, and *Staphylococcus* in LN tanks (Fountain et al. 1997; Ramin et al. 2014).

A considerable number of bacterial genera have known physiological characteristics that are likely to improve their survival under the specific conditions in the LN storage tanks. It has recently been shown that psychrophilic and cryotolerant bacteria attain higher culturability after freezing than their mesophilic relatives (Bajerski et al. 2018). The *Janthinobacterium* spp. which were observed in the LN tanks by amplicon sequencing are known to tolerate low temperatures, ultraviolet radiation and other environmental stressors (Mojib et al. 2013), similar to the psychrophilic *Pedobacter glacialis* (Qiu et al. 2014) and many *Flavobacterium* spp. that were related to psychrophilic strains forming microbial mats in Antarctic lakes (e.g., *Flavobacterium psychrolimnae*; Van Trappen et al. 2005). Also, *Pseudomonas psychrophila* has been isolated from a cold room for food storage (Yumoto et al. 2001). Aside from adaptations to low temperatures, the formation of endospores provides a means for the survival of freezing (Shimkets 2013) and hence explains the occurrence of *Bacillus* spp. in the LN tanks (Fountain et al. 1997; Ramin et al. 2014).

The high relative abundance of *Elizabethkingia, Empedobacter*, and *Janthinobacterium* in debris samples of one individual LN storage tank and the taxonomic affiliation of the detected species points toward a specific, and highly concentrated, source for these bacteria. The LN storage tank investigated has been used to store bacterial strains in sealed glass capillaries for 27 years. Therefore, the most probable source of bacteria found in the debris of the tank is the cryostored biomaterial itself and the breakage of some of the capillaries stored. Among the bacteria stored in the tank, *Janthinobacterium spp.* (e. g., *Janthinobacterium lividum)* were also found in the amplicon sequences. Since *Elizabethkingia* and *Empedobacter* include some pathogenic species (Table S5) (Teo et al. 2014), our results indicate that additional precautions to prevent the rupture of glass capillaries and a leakage of their contents into the LN tank are certainly warranted. The fish pathogens *Flavobacterium succinicans* and *Flavobacterium psychrophilum* were exclusively found in samples from a LN tank in which fish samples were stored in glass flasks, and thus might originate from the cryopreserved material.

### General recommendations

Our study showed that the bacterial load in LN storage devices is often low or not detectable (Table 1). Cell counts and DNA contents of the LN samples themselves were in the range of the negative controls and hence at the detection limit of both methods used. By contrast, several samples from the ice accumulating underneath the tank lids and along the rim, as well as debris at the tank bottom, contained microorganism in detectable amounts. The abundance of microorganism was related to the characteristics of the biobanks and sample types. While the majority of species do not represent a threat for human health, they have the potential to contaminate stored sample materials. However, these species have not been reported from stored research samples. Some of the bacterial species detected are also known opportunistic pathogens and hence may cause problems in immunosuppressed patients. Microorganism can influence follow-up applications, as it was suggested for *Stenotrophomonas maltophilia* suppressing fertilization (Bielanski et al. 2003). Clearly, the use of well-sealed sample containers (thermally sealed straws, glass capillaries) should efficiently reduce the microbial entry in the LN storage tanks. Glass-based containers may break and in some instances leading to contamination of the tank proper as shown in the present study. Therefore, a second, outer protective container might be advisable in these cases. Based on our results, the reduction of ice formation in the tanks, avoiding physical contact with the ice layers, as well as improved SOPs for the preparation of samples, for cryopreservation and sample containment, would further improve the safe storage of biological samples in LN tanks.

**Table 1:** Summary table concluding the main results of the study. The microbial load was very small. Cell and bacterial 16S rRNA gene copy numbers in the LN phase were below the detection limit. Small numbers of bacteria of up to 10^4^ cells per ml were detected in the ice phase formed underneath the lids or accumulated at the bottom

Sampled phase	Cells and gene copies	Cells or gene copies	Effect of					PCR detection*		
	N_p < 0.5_/ N	N_p < 0.5_/ N	storage time	air conditioning supply	Opening frequency	Institute	Storage phase	Human	Mycoplasma	fungi
LN liquid phase	0/13	0/13	no	no	no	no	no	0/13	0/13	0/13
Ice rim/lid	2/18	8/18	yes	yes	yes	yes	no	0/18	3/18	3/18
debris	1/3	1/3				yes	na	1/3	1/3	1/3

*N*, number of samples; *N*_*p* < 0.5_, number of samples above the detection limit of the negative control; *yes*, detected; *no*, not detected; *na*, not applicable; *, detected in at least 2 out of 3 replicates

## Electronic supplementary material

ESM 1 (PDF 1911 kb)

## References

[CR1] Amir A, McDonald D, Navas-Molina JA, Kopylova E, Morton JT, Zech Xu Z, Kightley EP, Thompson LR, Hyde ER, Gonzalez A, Knight R (2017) Deblur rapidly resolves single-nucleotide community sequence patterns. MSYSTEMS 2(2). 10.1128/mSystems.00191-16 10.1128/mSystems.00191-16 PMC5340863 28289731

[CR2] Bach EM, Williams RJ, Hargreaves SK, Yang F, Hofmockel KS (2018). Greatest soil microbial diversity found in micro-habitats. Soil Biol Biochem.

[CR3] Bailey TL, Gribskov M (1998). Combining evidence using p-values: application to sequence homology searches. Bioinformatics(Oxford, England).

[CR4] Bajerski F, Stock J, Hanf B, Darienko T, Heine-Dobbernack E, Lorenz M, Naujox L, Keller ERJ, Schumacher HM, Friedl T, Eberth S, Mock H-P, Kniemeyer O, Overmann J (2018). ATP content and cell viability as indicators for cryostress across the diversity of life. Front Psychol.

[CR5] Bielanski A, Bergeron H, Lau PCK, Devenish J (2003). Microbial contamination of embryos and semen during long term banking in liquid nitrogen. Cryobiology.

[CR6] Bielanski A, Nadin-Davis S, Sapp T, Lutze-Wallace C (2000). Viral contamination of embryos cryopreserved in liquid nitrogen. Cryobiology.

[CR7] Bielanski A, Vajta G (2009). Risk of contamination of germplasm during cryopreservation and cryobanking in IVF units. Hum Reprod.

[CR8] Bokulich Nicholas A, Subramanian Sathish, Faith Jeremiah J, Gevers Dirk, Gordon Jeffrey I, Knight Rob, Mills David A, Caporaso J Gregory (2012). Quality-filtering vastly improves diversity estimates from Illumina amplicon sequencing. Nature Methods.

[CR9] Buell CR, Joardar V, Lindeberg M, Selengut J, Paulsen IT, Gwinn ML, Dodson RJ, Deboy RT, Durkin AS, Kolonay JF (2003). The complete genome sequence of the Arabidopsis and tomato pathogen *Pseudomonas syringae* pv. *tomato* DC3000. Proc Natl Acad Sci USA..

[CR10] Caporaso JG, Kuczynski J, Stombaugh J, Bittinger K, Bushman FD, Costello EK, Fierer N, Peña AG, Goodrich JK, Gordon JI (2010). QIIME allows analysis of high-throughput community sequencing data. Nat Methods.

[CR11] Chao A, Jost L (2012). Coverage-based rarefaction and extrapolation: standardizing samples by completeness rather than size. Ecology.

[CR12] Chatterjee A, Saha D, Niemann H, Gryshkov O, Glasmacher B, Hofmann N (2017). Effects of cryopreservation on the epigenetic profile of cells. Cryobiology.

[CR13] Drexler HG, Uphoff CC (2002). *Mycoplasma* contamination of cell cultures: incidence, sources, effects, detection, elimination, prevention. Cytotechnology.

[CR14] Fountain D, Ralston M, Higgins N, Gorlin JB, Uhl L, Wheeler C, Antin JH, Churchill WH, Benjamin RJ (1997). Liquid nitrogen freezers: a potential source of microbial contamination of hematopoietic stem cell components. Transfusion.

[CR15] Gossner MM, Lewinsohn TM, Kahl T, Grassein F, Boch S, Prati D, Birkhofer K, Renner SC, Sikorski J, Wubet T (2016). Land-use intensification causes multitrophic homogenization of grassland communities. Nature.

[CR16] Hartig F (2017) DHARMa: residual diagnostics for hierarchical (multi-level/mixed) regression models. R package version 0.1. 5.

[CR17] Herberich Esther, Sikorski Johannes, Hothorn Torsten (2010). A Robust Procedure for Comparing Multiple Means under Heteroscedasticity in Unbalanced Designs. PLoS ONE.

[CR18] Kabiri L, Alum A, Rock C, McLain JE, Abbaszadegan M (2013). Isolation of *Bacteroides* from fish and human fecal samples for identification of unique molecular markers. Can J Microbiol.

[CR19] Kato Y, Asahara M, Goto K, Kasai H, Yokota A (2008). *Methylobacterium persicinum* sp. nov., *Methylobacterium komagatae* sp. nov., *Methylobacterium brachiatum* sp. nov., *Methylobacterium tardum* sp. nov. *and Methylobacterium gregans* sp. nov., isolated from freshwater. Int J Syst Evol Microbiol..

[CR20] Katoh K, Standley DM (2013). MAFFT Multiple Sequence Alignment Software Version 7: Improvements in Performance and Usability. Mol Biol Evol.

[CR21] Kelly DP, McDonald IR, Wood AP (2014) The Family *Methylobacteriaceae*. In: Rosenberg E, DeLong EF, Lory S, Stackebrandt E, Thompson F (eds) The Prokaryotes: *Alphaproteobacteria *and *Betaproteobacteria*. Springer, Berlin Heidelberg, pp 313–340

[CR22] Klappenbach JA, Dunbar JM, Schmidt TM (2000). rRNA operon copy number reflects ecological strategies of bacteria. Appl Environ Microbiol.

[CR23] Knierim D, Menzel W, Winter S (2017). Analysis of the complete genome sequence of euphorbia ringspot virus, an atypical member of the genus *Potyvirus*. Arch Virol.

[CR24] Kovaleva J, Degener JE, van der Mei HC (2014). *Methylobacterium* and its role in health care-associated infection. J Clin Microbiol.

[CR25] Kulakov LA, McAlister MB, Ogden KL, Larkin MJ, O'Hanlon JF (2002). Analysis of bacteria contaminating ultrapure water in industrial systems. Appl Environ Microbiol.

[CR26] Kuznetsova A, Brockhoff PB, Christensen RHB (2015) Package ‘lmerTest’. R package version 2(0)

[CR27] La Duc MT, Dekas A, Osman S, Moissl C, Newcombe D, Venkateswaran K (2007). Isolation and characterization of bacteria capable of tolerating the extreme conditions of clean room environments. Appl Environ Microbiol.

[CR28] Lauterboeck L, Gryshkov O, Hofmann N, Glasmacher B, Sciences IIRWoCAiL (2016) Importance of controlled ice formation for efficient cell biobanking. Refrigeration Sci Technol 84-90

[CR29] McArdle BH, Anderson MJ (2001). Fitting multivariate models to community data: a comment on distance-based redundancy analysis. Ecology.

[CR30] McMurdie PJ, Holmes S (2013). phyloseq: an R package for reproducible interactive analysis and graphics of microbiome census data. PLoS One.

[CR31] Mijnendonckx K, Provoost A, Ott CM, Venkateswaran K, Mahillon J, Leys N, Van Houdt R (2013). Characterization of the survival ability of *Cupriavidus metallidurans* and *Ralstonia pickettii* from space-related environments. Microb Ecol.

[CR32] Moissl-Eichinger C, Rettberg P, Pukall R (2012). The first collection of spacecraft-associated microorganisms: a public source for extremotolerant microorganisms from spacecraft assembly clean rooms. Astrobiology.

[CR33] Mojib N, Farhoomand A, Andersen DT, Bej AK (2013). UV and cold tolerance of a pigment-producing Antarctic *Janthinobacterium* sp. Ant5-2. Extremophiles.

[CR34] Morris GJ (2005). The origin, ultrastructure, and microbiology of the sediment accumulating in liquid nitrogen storage vessels. Cryobiology.

[CR35] Overmann J, Rosenberg E, DeLong EF, Lory S, Stackebrandt E, Thompson F (2013). Principles of enrichment, isolation, cultivation, and preservation of prokaryotes. The prokaryotes: prokaryotic biology and symbiotic associations.

[CR36] Overmann J (2015). Significance and future role of microbial resource centers. Syst Appl Microbiol.

[CR37] Overmann J, Abt B, Sikorski J (2017). Present and future of culturing bacteria. Annu Rev Microbiol.

[CR38] Overmann J, Smith D (2017) Microbial resource centers contribute to bioprospecting of bacteria and filamentous microfungi bioprospecting. Springer, pp 51-79

[CR39] Pascual J, von Hoermann C, Rottler-Hoermann A-M, Nevo O, Geppert A, Sikorski J, Huber KJ, Steiger S, Ayasse M, Overmann J (2017). Function of bacterial community dynamics in the formation of cadaveric semiochemicals during in situ carcass decomposition. Environ Microbiol.

[CR40] Piepho H-P (2004). An algorithm for a letter-based representation of all-pairwise comparisons. J Comput Graph Stat.

[CR41] Price MN, Dehal PS, Arkin AP (2010). FastTree 2 – Approximately maximum-likelihood trees for large alignments. PLoS One.

[CR42] Qiu X, Qu Z, Jiang F, Ren L, Chang X, Kan W, Fang C, Peng F (2014). *Pedobacter huanghensis* sp. nov. and *Pedobacterglacialis* sp. nov., isolated from Arctic glacier foreland. Int J Syst Evol Microbiol..

[CR43] Quast C, Pruesse E, Yilmaz P, Gerken J, Schweer T, Yarza P, Peplies J, Glöckner FO (2013). The SILVA ribosomal RNA gene database project: improved data processing and web-based tools. Nucleic Acids Res.

[CR44] R-Core-Team (2017) R: A language and environment for statistical computing. PUblisher. http://www.R-project.org/. Accessed 18 Jan 2018

[CR45] Rainey FA, Ward-Rainey NL, Janssen PH, Hippe H, Stackebrandt E (1996). *Clostridium paradoxum* DSM 7308^T^ contains multiple 16S rRNA genes with heterogeneous intervening sequences. Microbiology.

[CR46] Rall WF, Fahy GM (1985). Ice-free cryopreservation of mouse embryos at−196 C by vitrification. Nature.

[CR47] Ramin M, Bürger A, Hörlein A, Kerkau D, von Walcke-Wulffen V, Nicklas W, Schenkel J (2014). Stability of cryopreserved samples of mutant mice. Biopreserv Biobank.

[CR48] Reimer LC, Vetcininova A, Carbasse JS, Söhngen C, Gleim D, Ebeling C, Overmann J (2018). BacDive in 2019: bacterial phenotypic data for high-throughput biodiversity analysis. Nucleic Acids Res.

[CR49] Rittinghaus T, Glasmacher B (2018). Is freezer cooling rate equal to sample cooling rate?. Cryobiology.

[CR50] Rognes T, Flouri T, Nichols B, Quince C, Mahé F (2016). VSEARCH: a versatile open source tool for metagenomics. PeerJ.

[CR51] Ryan KJ, Ray CG (2004). Medical microbiology. McGraw Hill.

[CR52] Salter S, Cox MJ, Turek EM, Calus ST, Cookson WO, Moffatt MF, Turner P, Parkhill J, Loman N, Walker AW (2014). Reagent contamination can critically impact sequence-based microbiome analyses. bioRxiv.

[CR53] Schüngel M, Smith D, Bizet C, Stackebrandt E, Consortium M (2014). The role of the European microbial resource research infrastructure project. Enliven: Microb Microbial Tech.

[CR54] Shimkets LJ, Rosenberg E, DeLong EF, Lory S, Stackebrandt E, Thompson F (2013). Prokaryotic Life Cycles. The prokaryotes: prokaryotic communities and ecophysiology.

[CR55] Stackebrandt E, Rosenberg E, DeLong EF, Lory S, Stackebrandt E, Thompson F (2014). The Family *Propionibacteriaceae*: Genera other than *Propionibacterium*. The prokaryotes: actinobacteria.

[CR56] Stock J, Mock H-P, Senula A, Nagel M (2018) Arabidopsis - A model to elucidate complex stress response mechanism during cryopreservation. Acta Hortic. Accessed 27 Aug 2018

[CR57] Team, Q. d. (2016-2018 https://qiime2.org/). "https://qiime2.org/."

[CR58] Tedder RS, Zuckerman MA, Brink NS, Goldstone AH, Fielding A, Blair S, Patterson KG, Hawkins AE, Gormon AM, Heptonstall J, Irwin D (1995). Hepatitis B transmission from contaminated cryopreservation tank. Lancet.

[CR59] Teo J, Tan SY-Y, Liu Y, Tay M, Ding Y, Li Y, Kjelleberg S, Givskov M, Lin RTP, Yang L (2014). Comparative genomic analysis of malaria mosquito vector-associated novel pathogen *Elizabethkingia anophelis*. Genome Biol Evol.

[CR60] Uphoff CC, Drexler HG (2002). Comparative PCR analysis for detection of mycoplasma infections in continuous cell lines. In Vitro Cell. Dev Biol Animal.

[CR61] Uphoff CC, Lange S, Denkmann SA, Garritsen HSP, Drexler HG (2015). Prevalence and characterization of murine leukemia virus contamination in human cell lines. PLoS One.

[CR62] Van Trappen S, Vandecandelaere I, Mergaert J, Swings J (2005). *Flavobacterium fryxellicola* sp. nov. and *Flavobacterium psychrolimnae* sp. nov., novel psychrophilic bacteria isolated from microbial mats in Antarctic lakes. Int J Syst Evol Microbiol.

[CR63] Wang K, Lu W, Tu Q, Ge Y, He J, Zhou Y, Gou Y, Van Nostrand JD, Qin Y, Li J, Zhou J, Li Y, Xiao L, Zhou X (2016) Preliminary analysis of salivary microbiome and their potential roles in oral lichen planus. Sci Rep 6:22943. 10.1038/srep22943https://www.nature.com/articles/srep22943#supplementary-information. Accessed 26 Sept 2017 10.1038/srep22943 PMC4785528 26961389

[CR64] Yilmaz P, Parfrey LW, Yarza P, Gerken J, Pruesse E, Quast C, Schweer T, Peplies J, Ludwig W, Glöckner FO (2013). The SILVA and “all-species living tree project (LTP)” taxonomic frameworks. Nucleic Acids Res.

[CR65] Yumoto I, Kusano T, Shingyo T, Nodasaka Y, Matsuyama H, Okuyama H (2001). Assignment of *Pseudomonas* sp. strain E-3 to *Pseudomonas psychrophila* sp. nov., a new facultatively psychrophilic bacterium. Extremophiles.

