# Supplementary material for: Factors determining microbial colonization of liquid nitrogen storage tanks used for archiving biological samples
Source: Appl Microbiol Biotechnol. 2019 Nov 28;104(1):131–44. doi: 10.1007/s00253-019-10242-1 (PMC6942587; doi:10.1007/s00253-019-10242-1)
Supplement: Supplementary file 1 — (PDF 1911 kb) [file 253_2019_10242_MOESM1_ESM.pdf]

Journal/Research topic: Applied microbiology and biotechnology / Biotechnological products and process engineering

Title: Factors determining microbial colonization of liquid nitrogen storage tanks used for archiving biological samples

Authors: F. Bajerski[1], A. Bürger[2], B. Glasmacher[3], E. R. J. Keller[4], K. Müller[5], K. Mühldorfer[5], M. Nagel[4], H. Rüdel[6], T. Müller[7] J. Schenkel[8*], & J. Overmann[1,9*#]
[1]Leibniz Institute DSMZ-German Collection of Microorganisms and Cell Cultures, Braunschweig, Germany
[2]Helmholtz Zentrum München, German Research Center for Environmental Health, Institute of Developmental Genetics, München, Germany
[3]Leibniz Universität Hannover, Institute for Multiphase Processes, Hannover, Germany
[4]Leibniz Institute of Plant Genetics and Crop Plant Research (IPK), Seeland, OT Gatersleben, Germany
[5]Leibniz Institute for Zoo and Wildlife Research (Leibniz-IZW), Berlin, Germany
[6]Fraunhofer Institute for Molecular Biology and Applied Ecology IME, Schmallenberg, Germany
[7]BioKryo GmbH, Sulzbach, Germany
[8]German Cancer Research Centre, Heidelberg, Germany
[9]Microbiology, Braunschweig University of Technology, Braunschweig, Germany

* Contributed equally

[#]**Corresponding author:**
Felizitas Bajerski
Leibniz Institute DSMZ-German Collection of Microorganisms and Cell Cultures
Inhoffenstraße 7B
38124 Braunschweig, Germany
Phone: +49 (0) 531-2616-352
Fax: +49 (0) 531-2616-418
E-mail: Felizitas.Bajerski@dsmz.de

Figure S1. Effect plots of a generalized linear model with Gaussian distribution. Effect of predictor variables on a response variable (cell numbers) after removing samples below the detection limit (leaving out institute E, J). Predictors: institute, sampled phase (phase), storage phase (storage), surrounding condition, (condition), number of openings (open_num), usage time (in_use). The variables storage material and storage device were included in the model as well, but their effect is redundant with the other variables. *, significant intercept.

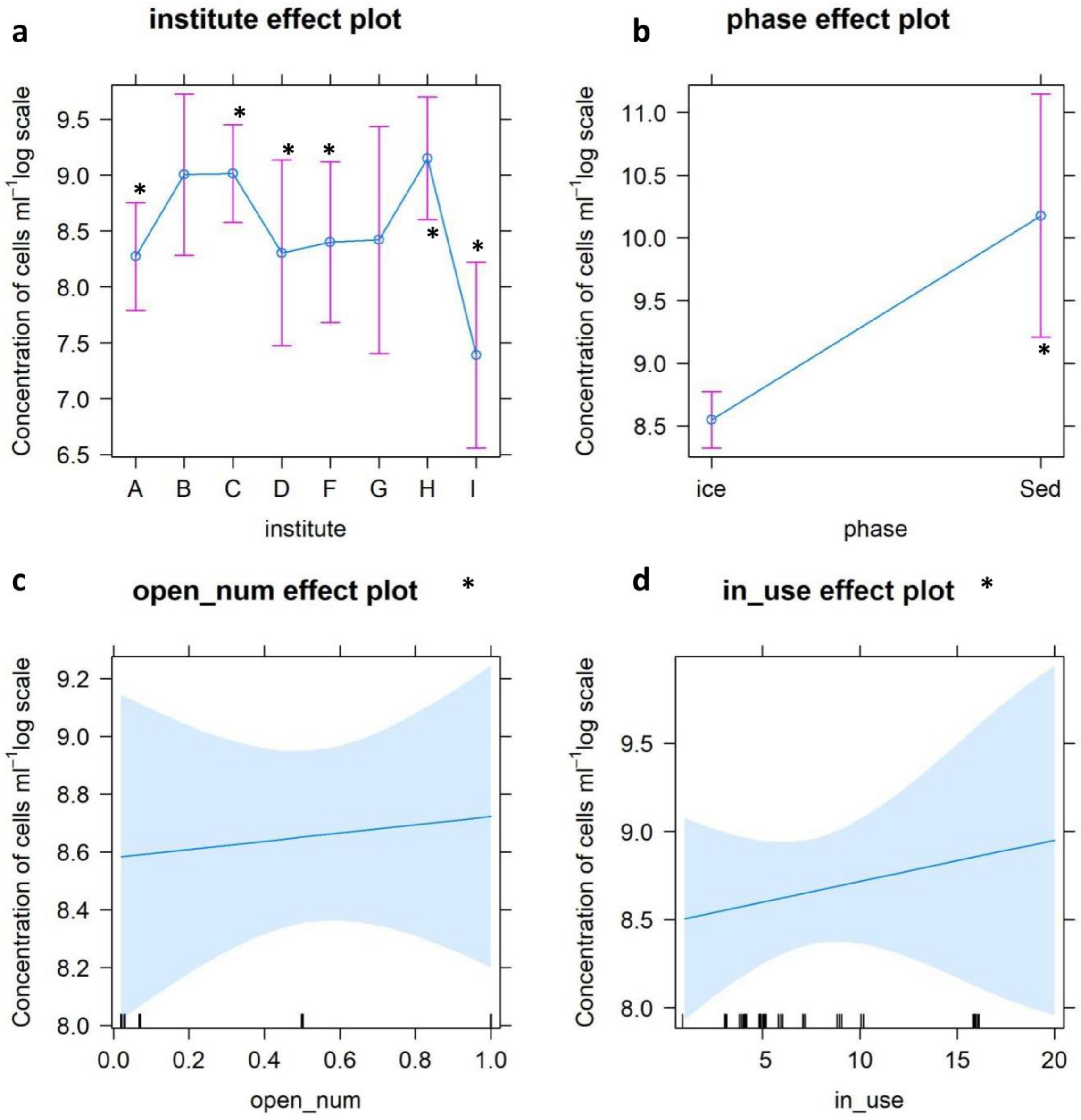

Figure S2. Relative abundance (%) of the 10 most abundant phyla in each liquid nitrogen storage tank including all samples. ID, identity number (Table S1).

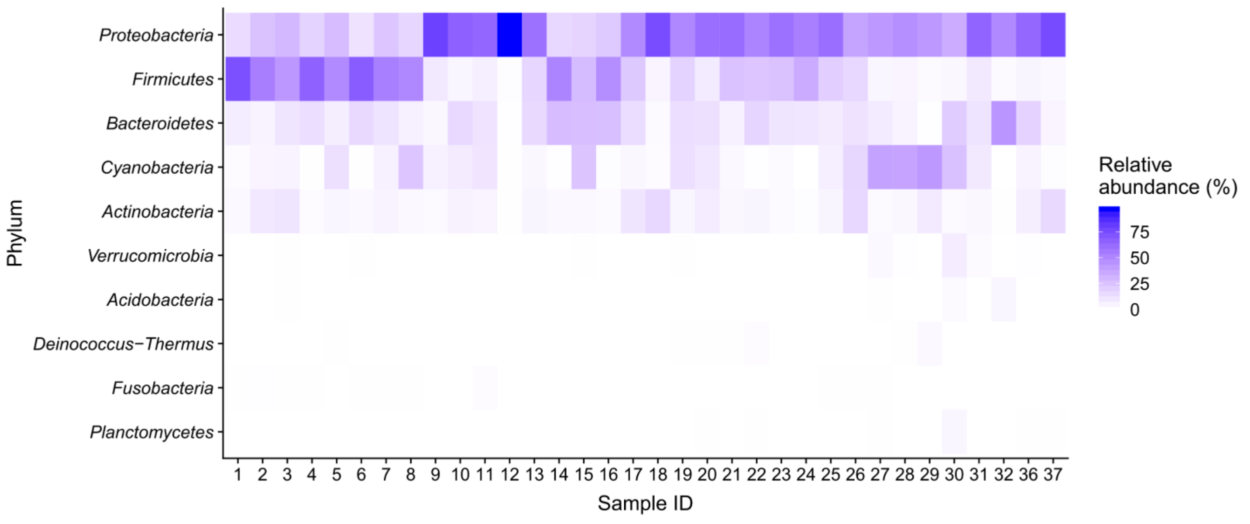

Figure S3. Morphology of detected green algae and diatoms . Cells of *Chlorella* spec. a) bright field color, b) autofluorescence (excitation 425 nm, emission 630 nm) of chlorophyll a. Diatoms c) pennate and d) centric. Scale bar, 10 µm.

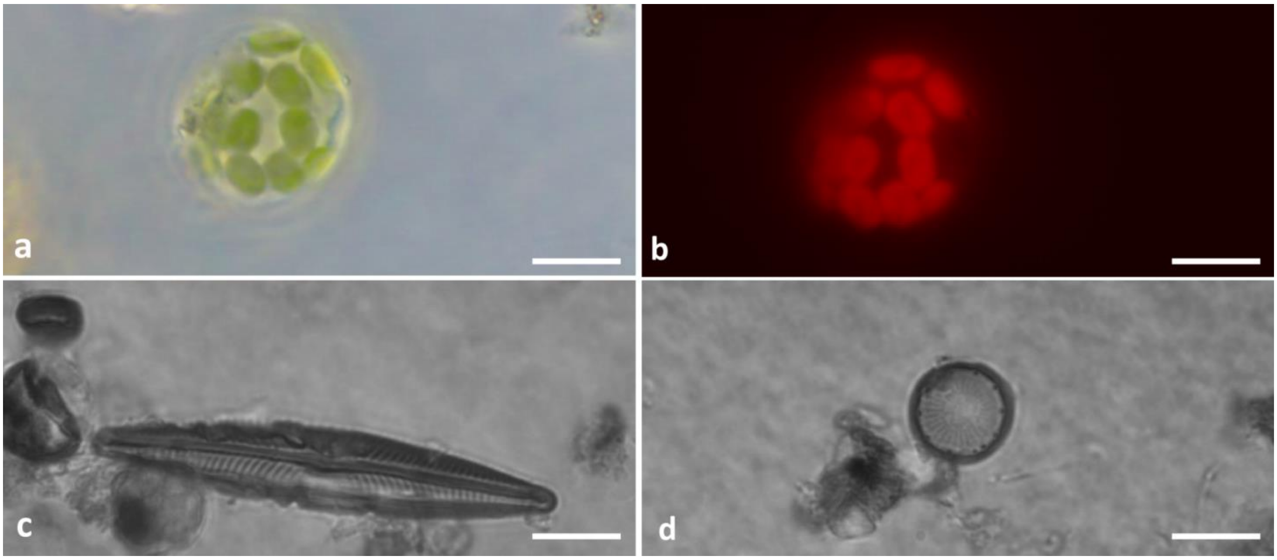

Figure S4. Abundance of sequences affiliated with the phylum Cyanobacteria (class Chloroplast, Cyanobacteria) in relation to the material that was stored in the liquid nitrogen (LN) storage tanks. The significance of differences of the abundance between the different groups of stored material were calculated (by ANOVA) and shown as compact letter display. NC, negative control; mio, microorganisms, plant_mix, plant and other eukaryotes; euk, eukaryotes (animal, human).

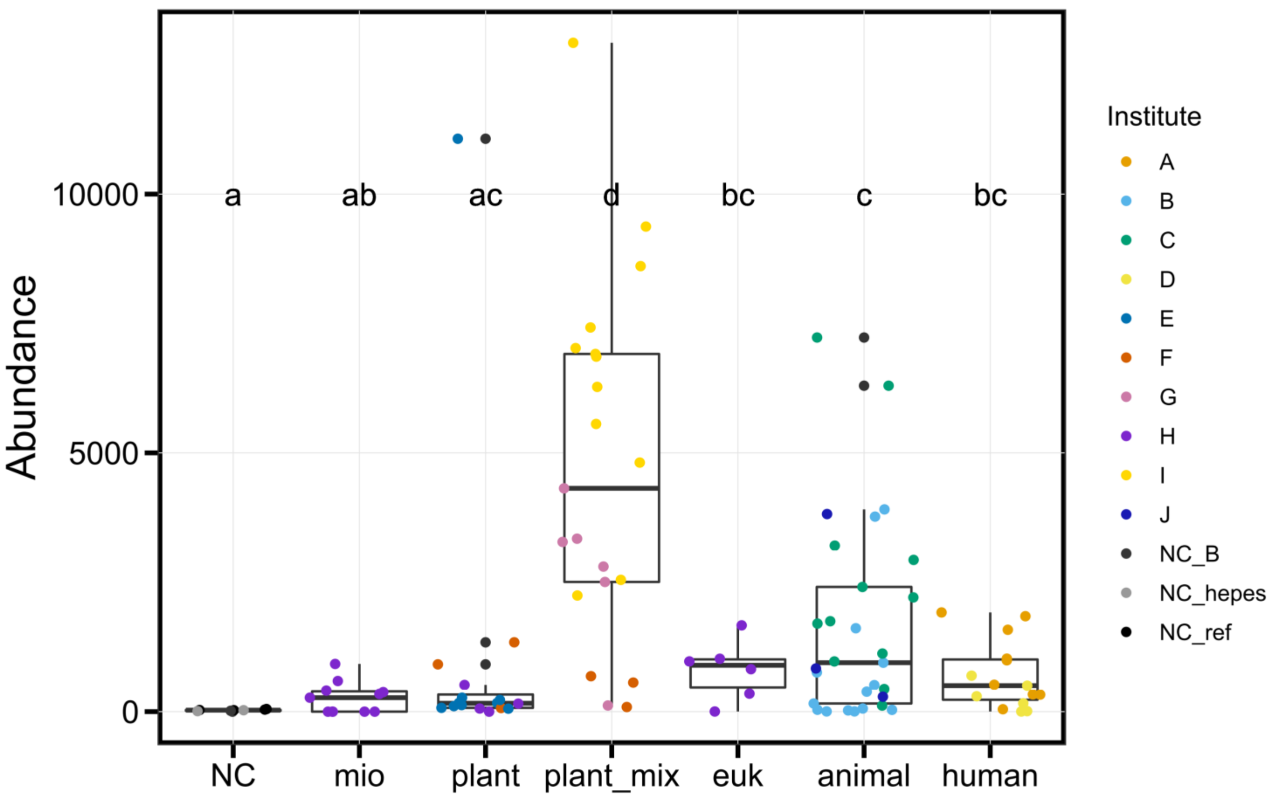

Figure S5. Overview of α- diversity measures; a)observed sequence variants, b) Chao1 and c) Shannon diversity index). The significance of differences of the α-diversity measures between the different institutes was determined by one-way-ANOVA with multiple comparisons of means using Tukey Contrasts (R package multcomp) and shown as compact letter display. A-J Identification letter of each institute. NC B, exemplary negative control of institute B; NC hepes/ NC_h, LN reference sample including HEPES buffer; NC ref/ NC_r, LN reference sample without HEPES buffer.

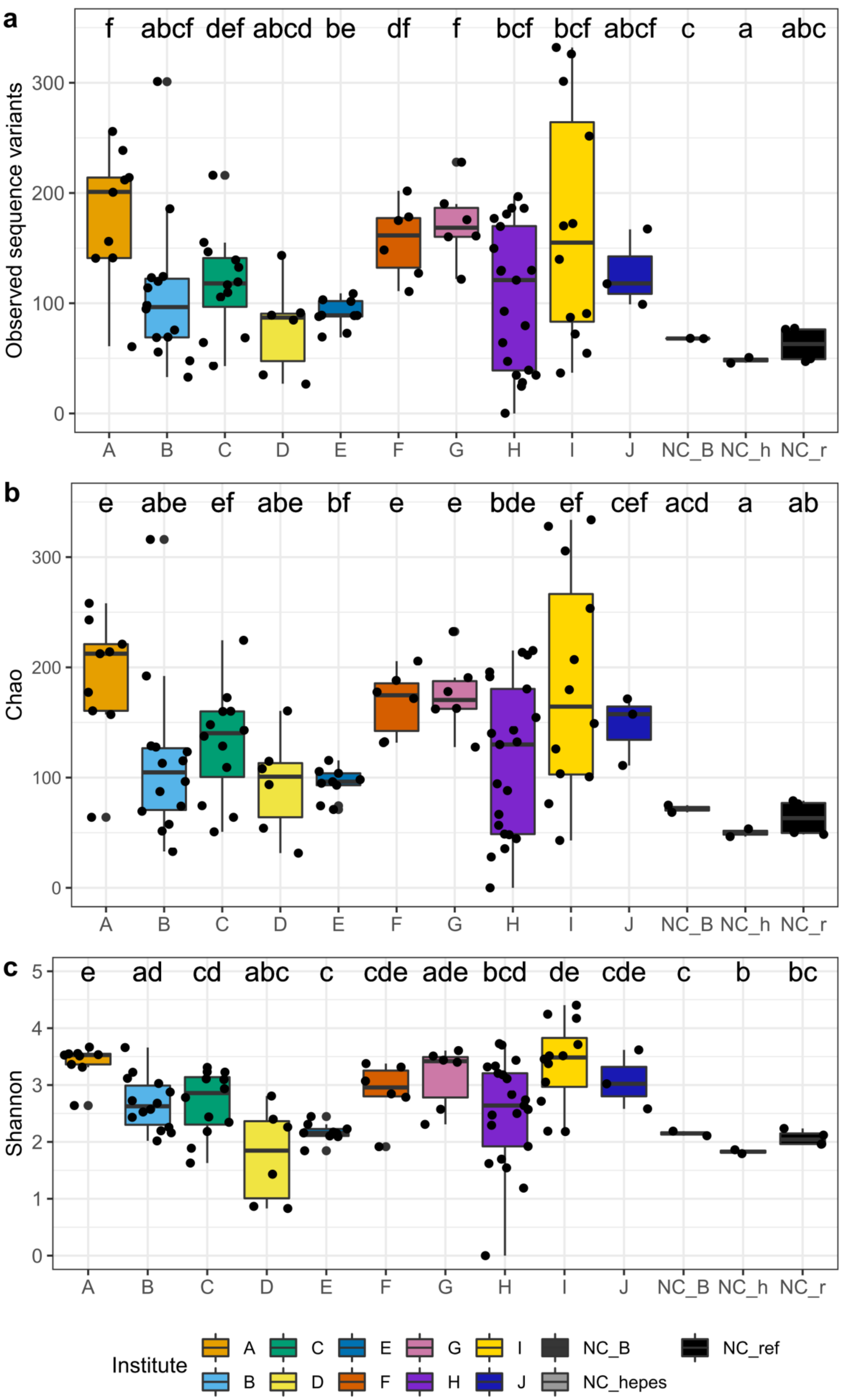

Figure S6. Bacterial community structure at species level analyzed with principal coordinate analysis (PcOA) based on on weighted unifrac distances. The ellipses are calculated as 95 % confidence intervals grouped by the sampled phase. The NC samples group with the LN samples according to species distribution. Shapes: Sampled phase; LN, liquid nitrogen; Deb, debris; NC, negative control. Colors: A-J, institutes; NC_B, exemplary negative control of institute B; NC_hepes, LN reference sample including HEPES buffer; NC_ref, LN reference sample without HEPES buffer.

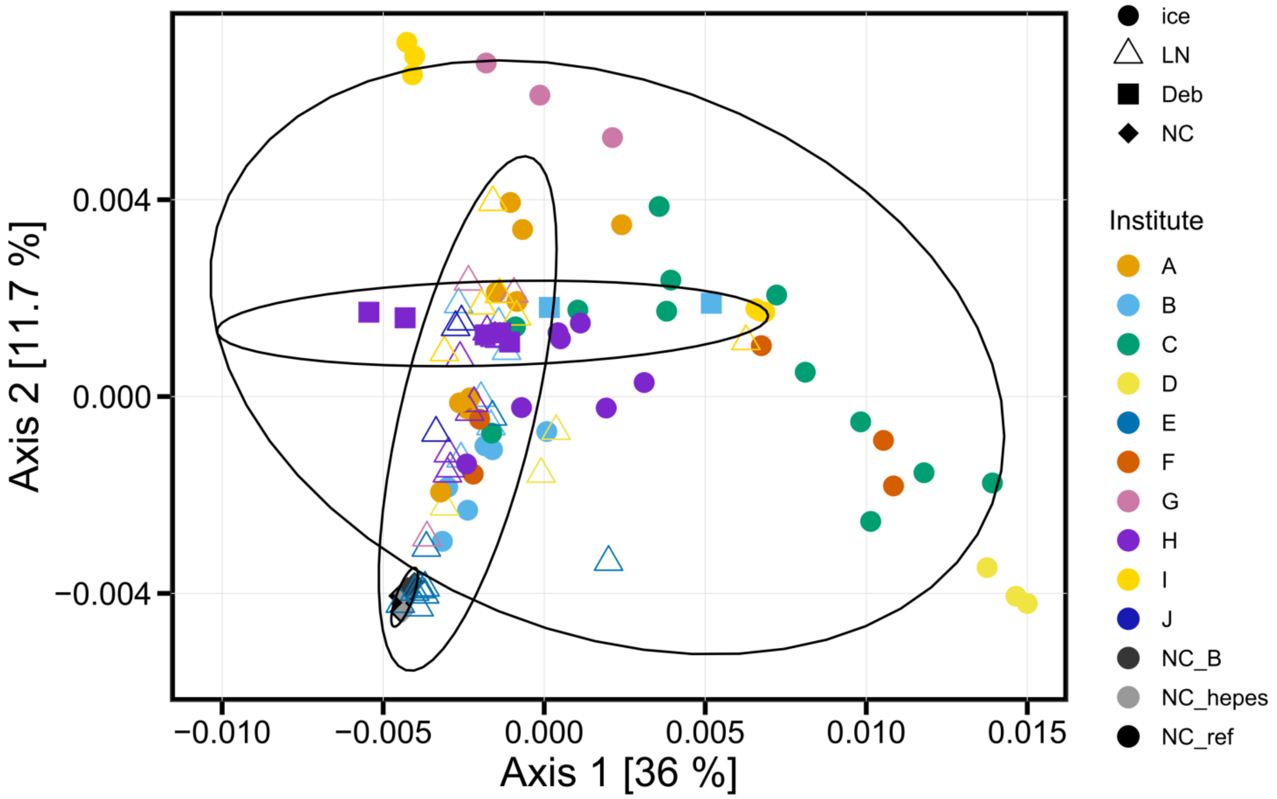

Figure S7. Relative abundance (%) of the 20 most abundant genera in each liquid nitrogen storage tank.

The second (right) y-axis indicates the affiliation of the respective genera to risk groups and wether they are listed by the “Zentrale Kommission für die Biologische Sicherheit” (in brackets: ZKBS). y, yes; n, no.

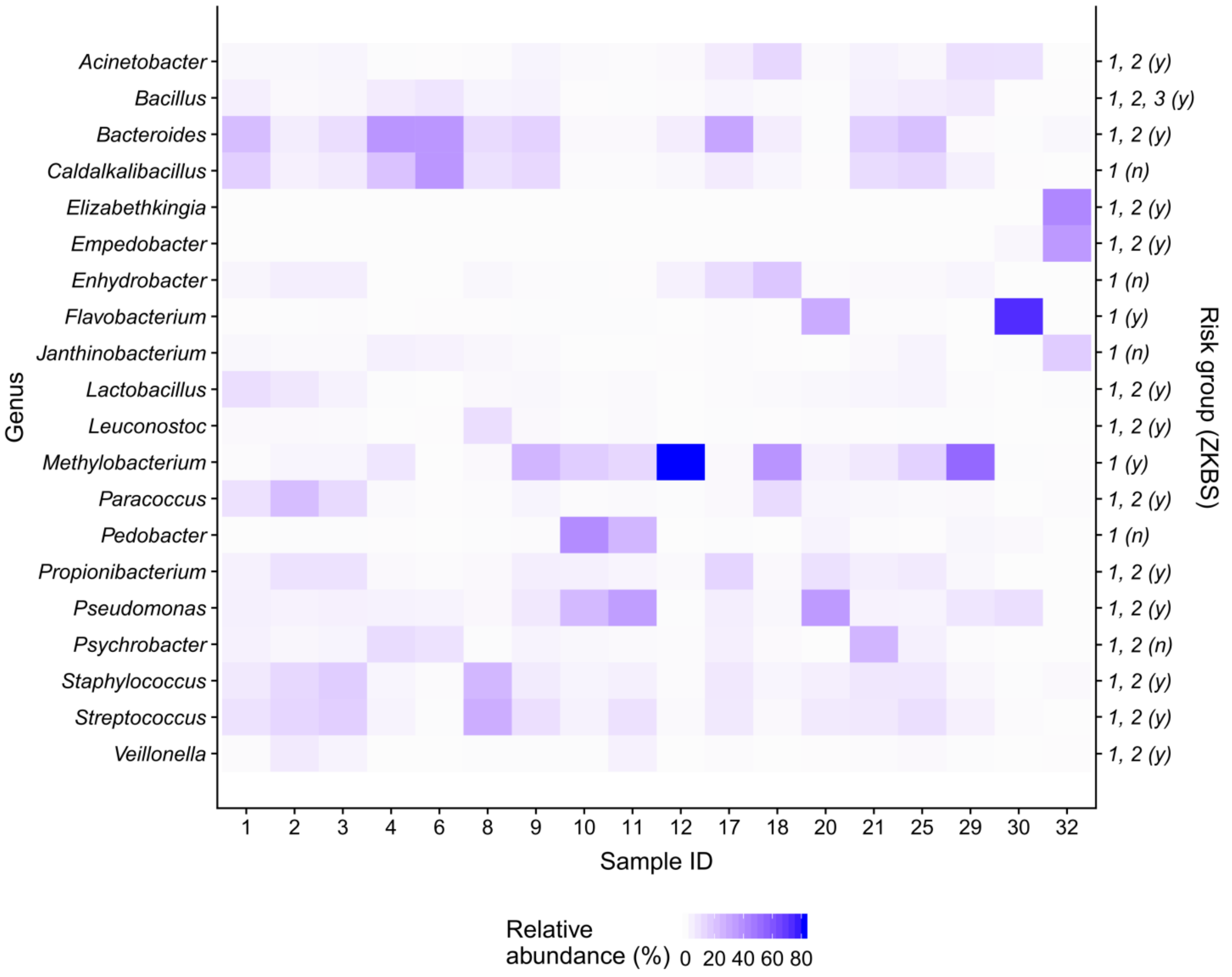

Table S1: Overview of the participating institutes (anonymized as A-J) and metadata of the liquid nitrogen (LN) storage tanks. ID, identification number; R, replicate; NC eq, negative controls of laboratory equipment; NC B, exemplary NC of institute B; NC HEPES, LN reference sample including HEPES buffer; NC ref, LN reference sample without HEPES buffer; Deb, Debris in the bottom of the LN storage tank; gN, gaseous phase of the LN storage tanks.

**Insti-tute**	**ID**	R	Sampled **phase**	**in use** (years)	Frequency of opening **open**	**Storage** phase	**storage device**	Stored material **samples**	Storage **condition**
A	1	3	ice	9	every second day	gN	bags (in metal cassettes)	human	filtered air supply
A	2	3	ice	4	daily	gN	bags	human	filtered air supply
A	3	3	ice	4	daily	gN	bags	human	filtered air supply
B	4	3	ice	7	every second day	LN	straws (IMV, sealed)	animal (rodent)	air exhaust
B	5	3	LN	7	every second day	LN	straws (IMV, sealed)	animal (rodent)	air exhaust
B	6	3	ice	1	every second day	LN	straws (IMV, sealed)	animal (rodent)	air exhaust
B	7	3	LN	1	every second day	LN	straws (IMV, sealed)	animal (rodent)	air exhaust
B	36	1	Deb	13	every second day	LN	straws (IMV, sealed)	animal (rodent)	air exhaust
B	37	1	Deb	30	every second day	LN	straws (IMV, sealed)	animal (rodent)	air exhaust
C	8	3	ice	6	every second week	gN	tubes	animal (rodent)	air exhaust
C	9	3	ice	5	every second week	gN	tubes	animal (rodent)	air exhaust
C	10	3	ice	3	every second day	gN	tubes	animal (rodent)	air exhaust
C	11	3	ice	4	every second day	gN	tubes	animal (rodent)	air exhaust
D	12	3	ice	5	once a month	gN	bags	human	filtered air supply
D	13	3	LN	5	once a month	gN	straws (minitubes, Fa. MTG)	human	filtered air supply
E	14	3	LN	24	every second week	LN	tubes	plant	air supply
E	15	3	LN	4	every second day	LN	tubes	plant	air supply
E	16	3	LN	3	every second week	LN	tubes	plant	air supply
F	17	3	ice	3	seldom	LN	tubes	plant	filtered air supply and air exhaust

F	18	3	ice	5	seldom	mix	mix (tubes, bags)	mix (plants, human)	filtered air supply and air exhaust
G	19	3	LN	17	once a week	LN	tubes	mix (human, animal (rodent, pig, monkey), plant)	filtered air supply
G	20	3	ice	10	every second day	gN	tubes	mix (human, animal (rodent, pig, monkey), plant)	hall
H	21	3	ice	16	daily	mix	mix (tubes, glass capillaries, glass vials)	mix (bacteria, phages, fungi)	air supply and air exhaust
H	22	3	LN	16	daily	mix	mix (tubes, glass capillaries, glass vials)	mix (bacteria, phages, fungi)	air supply and air exhaust
H	23	3	LN	7	once a week	LN	tubes	plant	air exhaust
H	24	1	ice	7	once a week	LN	tubes	plant	air exhaust
H	25	3	ice	5	every second day	mix	tubes	mix (human, animal)	air exhaust
H	26	3	LN	2	every second day	mix	tubes	mix (human, animal)	air exhaust
H	32	5	Deb	16	daily	mix	mix (tubes, glass capillaries, glass vials)	mix (bacteria, phages, fungi)	air supply and air exhaust
I	27	3	LN	16	daily	gN	container	mix (plant (leaves), animal (fish, mussels, dove egg))	filtered air supply and air exhaust
I	28	3	LN	16	daily	gN	container	mix (plant (leaves), animal (fish, mussels, dove egg))	filtered air supply and air exhaust
I	29	3	ice	16	daily	gN	container	mix (plant (leaves), animal (fish, mussels, dove egg))	filtered air supply and air exhaust
I	30	3	ice	16	daily	gN	mix (glass flasks, scintillation vials)	mix (plant (leaves), animal (fish, mussels, dove egg))	filtered air supply and air exhaust
J	31	3	LN	8	every second day	mix	mix (straws, tubes, box, container)	animal	air supply
NC ref	33	4	NC	NC	NC	NC	NC	NC	NC
NC hep	34	2	NC	NC	NC	NC	NC	NC	NC
NC B	35	2	NC	NC	NC	NC	NC	NC	NC
NC eq	0	15	NC						

Table S2: Primer and protocols used for specific amplification

Primer	Sequence (5´-)	Literature	Protocol
**Bacterial 16 S rRNA gene**			
8 F	AGAGTTTGATCCTGGCTCAG	(Turner et al. 1999)	95°C 10 min, 34x (94°C 30 sec, 55°C 30 sec, 72°C 2 min 10 sec), 72°C 10 min)
1492 R	GGTTACCTTGTTACGACTT		
**Bacterial 16 S r RNA gene, V3 Region**			
341 F	CCTACGGGWGGCWGCAG	(Muyzer et al. 1993)	95°C 5 min, 50x (95°C 10 sec, 63°C 15 sec, 72°C 10 sec))
515 R	CCGCGGCTGCTGGCAC	(Cho et al. 1996)	
**Mycoplasma 16 S rRNA gene**			
5´ primer mix	cgc ctg agt agt acg ttc gc<br>cgc ctg agt agt acg tac gc<br>tgc ctg ggt agt aca ttc gc<br>tgc ctg agt agt aca ttc gc<br>cgc ctg agt agt atg ctc gc<br>cac ctg agt agt atg ctc gc<br>cgc ctg ggt agt aca ttc gc	(Uphoff and Drexler 2002; Uphoff and Drexler 2013)	1x (96°C 2 min, 65°C 1 min, 72°C 1 min), 35x (94°C 4 sec, 65°C 8 sec, 72°C 16 sec + 2 sec extension time per cycle)
primer mix	gcg gtg tgt aca aga ccc ga<br>gcg gtg tgt aca aaa ccc ga<br>gcg gtg tgt aca aac ccc ga		
**Line 1 (mouse, rabbit, rat, human) 290 bp**			
Line 1 F	CCATGCTCATSGATTGG	(Dobigny et al. 2004)	94°C 10 min, 34x (94°C 30 sec, 55°C 30 sec, 72°C 1 min), 72°C 10 min)
Line 1 R	ATTCTRTTCCATTGGTCTA		
**Fungi ITS**			
ITS1F 81735-1756 (18S)	CTTGGTCATTTAGAGGAAGTAA	(Gardes and Bruns 1993)	94°C 10 min, 32x (94°C 45 sec, 54°C 45 sec, 72°C 2 min), 72°C 10 min)
ITS4 LSU 60-41	TCCTCCGCTTATTGATATGC	(White TJ 1990)	

Cho J-C, Lee D-H, Cho Y-C, Cho J-C, Kim S-J (1996) Direct extraction of DNA from soil for amplification of 16S rRNA gene sequences by polymerase chain reaction. The Journal of Microbiology 34(3):229-235

Dobigny G, Ducroz J-F, Robinson TJ, Volobouev v (2004) Cytogenetics and Cladistics. Systematic Biology 53(3):470-484 doi:10.1080/10635150490445698

Gardes M, Bruns TD (1993) ITS primers with enhanced specificity for basidiomycetes - application to the identification of mycorrhizae and rusts. Molecular Ecology 2(2):113-118 doi:10.1111/j.1365-294X.1993.tb00005.x

Muyzer G, de Waal EC, Uitterlinden AG (1993) Profiling of complex microbial populations by denaturing gradient gel electrophoresis analysis of polymerase chain reaction-amplified genes coding for 16S rRNA. Applied and Environmental Microbiology 59(3):695-700

Turner S, Pryer KM, Miao VPW, Palmer JD (1999) Investigating Deep Phylogenetic Relationships among Cyanobacteria and Plastids by Small Subunit rRNA Sequence Analysis1. Journal of Eukaryotic Microbiology 46(4):327-338 doi:10.1111/j.1550-7408.1999.tb04612.x

Uphoff CC, Drexler HG (2002) Comparative PCR analysis for detection of mycoplasma infections in continuous cell lines. In Vitro Cellular & Developmental Biology-Animal 38(2):79-85

Uphoff CC, Drexler HG (2013) Detection of Mycoplasma Contaminations. In: Helgason CD, Miller CL (eds) Basic Cell Culture Protocols. Humana Press, Totowa, NJ, pp 1-13

White TJ BT, Lee S, Taylor (1990) Amplification and direct sequencing of fungal ribosomal RNA genes for phylogenetics. . In: Innis MA, Gelfand DH SJ, White TJ (eds) PCR Protocols: a guide to methods and applications. Academic Press, New York, USA, pp 315–322

Table S3. Calculation of DNA content and cell counts and PCR success to determine the microbial load of liquid nitrogen (LN) storage tanks. ID, identity number; A-J, Identification letter of each institute; N, number of replicates; SD, standard deviation; hu, human; fun, fungi; myc, *Mycoplasma*; PCR, polymerase chain reaction; NC eq, negative controls of laboratory equipment; hep, LN reference sample including HEPES buffer; ref, LN reference sample without HEPES buffer; Deb, Debris; nd, not determined; Nm, not measurable; code for PCR success: - negative, + positive, //, replicates ;* significant above mean of all NCs $p<0.05$; ** significant above mean of all NCs $p<0.01$.

ID	Institute	phase	N	cells per ml		copies per ml		DNA ng per µl		PCR Success		
				mean	SD	mean	SD	mean	SD	hu	fun	myc
1	A	ice	3	5.7E+03	5.3E+03	7.3E+04	3.1E+04	0.52	0.04	-/-/-	-/-/-	-/-/-
2	A	ice	3	3.9E+03*	1.5E+03	9.3E+04*	3.3E+04	0.57	0.03	-/-/-	-/-/-	-/-/-
3	A	ice	3	3.9E+03	1.9E+03	3.6E+04*	9.9E+03	0.51	0.07	-/-/-	-/-/-	-/-/-
4	B	ice	3	1.3E+04	7.6E+03	1.6E+06	2.8E+06	0.40	0.06	-/-/-	-/-/-	-/-/-
5	B	LN	3	5.7E+01	4.2E+01	2.0E+02	1.4E+02	0.43	0.03	-/-/-	-/-/+	+/-/-
6	B	ice	3	5.5E+03	5.5E+03	5.1E+04	6.1E+04	0.45	0.03	-/-/-	-/-/-	-/-/-
7	B	LN	3	5.7E+01	4.1E+01	9.0E+01	2.6E+01	0.38	0.05	-/-/-	-/-/-	+/-/-
36	B	Deb	1	nd	nm	9.5E+03	nm	0.68	nm	+	-	-
37	B	Deb	1	nd	nm	5.4E+03	nm	0.57	nm	-	-	-
8	C	ice	3	5.0E+03	2.0E+03	1.1E+04	7.6E+03	0.38	0.08	-/-/-	-/-/-	-/-/-
9	C	ice	3	5.4E+03	2.2E+03	3.6E+04	3.5E+04	0.42	0.12	-/-/-	-/+/-	-/-/-
10	C	ice	3	8.7E+03*	1.6E+03	1.1E+06	5.9E+05	1.34	0.88	-/-/-	+/+/+	-/-/-
11	C	ice	3	1.6E+04	8.1E+03	1.9E+06	9.7E+05	1.29	0.40	-/-/-	+/-/-	+//+
12	D	ice	3	4.2E+03	3.1E+03	7.9E+04**	8.2E+03	0.50	0.05	-/-/-	-/-/-	-/-/-
13	D	LN	3	3.0E+01	2.0E+01	3.3E+01	7.9E+00	0.36	0.06	-/-/-	-/-/-	-/-/-
14	E	LN	3	3.1E+01	2.0E+01	1.8E+01	5.9E+00	0.46	0.03	-/-/-	-/-/-	-/-/-
15	E	LN	3	2.2E+01	5.5E+00	3.6E+01	2.4E+01	0.48	0.07	-/-/-	-/-/-	-/-/-
16	E	LN	3	5.2E+01	4.2E+01	3.3E+01	7.2E+00	0.53	0.08	-/-/-	-/-/-	-/-/-
17	F	ice	3	5.0E+03*	4.3E+03	3.9E+03	1.9E+03	0.63	0.06	-/-/-	+/+/-	+/-/-
18	F	ice	3	3.9E+03	2.9E+03	1.9E+04*	5.5E+03	0.39	0.07	-/-/-	-/+/-	-/+/-
19	G	LN	3	2.2E+01	nm	1.0E+03	1.7E+03	0.44	0.06	-/-/-	-/-/-	-/-/-
20	G	ice	3	2.8E+03	2.5E+03	4.5E+04	7.0E+04	3.82	1.62	+/-/-	-/-/-	+/+/+
21	H	ice	3	9.2E+03	5.7E+03	6.6E+04	2.7E+04	0.38	0.03	-/-/-	-/-/-	-/-/-
22	H	LN	3	3.7E+01	1.9E+01	2.5E+03*	5.9E+02	0.66	0.26	-/-/-	-/-/-	-/+/-
23	H	LN	3	3.1E+01	8.6E+00	2.3E+01	5.2E+00	0.45	0.12	-/-/-	-/-/-	-/-/-
24	H	ice	1	nd	nm	2.9E+06	nm	0.37	nm	-	-	-
25	H	ice	3	9.2E+03*	3.2E+03	6.1E+04*	1.6E+04	0.37	0.01	-/-/-	-/-/-	-/-/-
26	H	LN	3	1.3E+01	1.2E+01	3.8E+01	5.3E+00	0.39	0.03	-/-/-	-/-/-	-/-/-
32	H	Deb	5	5.0E+04**	2.0E+04	7.9E+06*	8.0E+06	24.92	18.91	-/-/-/-/-	+/+/+/+/+	+/+/+/-/+
27	I	LN	3	2.2E+01	9.8E+00	2.8E+02	1.1E+02	1.04	0.59	-/-/-	-/-/-	-/-/-
28	I	LN	3	3.0E+01	1.6E+01	6.1E+02	5.1E+02	4.41	3.54	-/-/-	-/-/+	-/-/-
29	I	ice	3	1.5E+03*	3.2E+02	2.4E+05	1.0E+05	12.29	6.89	-/+/-	+/+/+	+/+/+
30	I	ice	3	2.6E+03	3.2E+02	nm	nm	13.40	6.02	-/-/-	-/-/-	+/-/-
31	J	LN	3	1.5E+01	1.6E+01	8.3E+01	1.3E+02	0.20	0.04	-/-/-	-/-/-	-/-/-
33	ref	NC	2	nd	nm	1.6E+01	6.8E+00	0.41	0.06	-/-/-/-	-/-/-/-	-/-/-/-
34	hep	NC	2	nd	nm	3.0E+02	1.4E+01	0.38	0.02	-/-	-/-	-/-
35	B	NC	2	1.8E+02	1.4E+02	1.2E+02	4.0E+01	0.17	0.08	-/-	-/-	-/-
0	eq	NC	13	1.9E+02	1.9E+02	1.1E+02	1.2E+02	0.32	0.09	-	-	-

Table S4: Summary statistics of the cell counts and copy number by the sampled phase determined in liquid nitrogen storage (LN) tanks. N, number of cases; sd, standard deviation; se, standard error; ci, 95%-confidence interval; Deb, Debris; NC B, exemplary negative control of institute B; NC B/hep/ref, exemplary NC of institute B/ LN reference sample including HEPES buffer/ LN reference sample without HEPES buffer.

	phase	N	counts	sd	se	ci
**cells ml^{-1}**	ice	49	6368	5059	723	1453
	LN	36	34	24	4	8
	Deb	6	43118	24431	9974	25639
	NC B	2	180	137	97	1233
**copies ml^{-1}**	ice	46	392768	944957	139326	280618
	LN	39	380	802	128	260
	Deb	8	5686196	6996596	2473670	5849301
	NC B/hep/ref	8	112	124	44	104

Table S5: Summary of the generalized linear model with Gaussian distribution. Effect of predictor variables (the institute, storage phase, surrounding condition, stored material, storage device, number of openings and the usage time) on a response variable (gene copies and cell numbers) after removing samples below the detection limit. The variables institutes, sampled phase (Debris), storage phase (LN = liquid nitrogen), surrounding condition (Air supply and air exhaust), number of openings, usage time predicted the occurrence of cells and copies. The effect of the variables storage material and storage device was redundant with the other variables. The estimate, standard error (SE), test statistic and p value of the model have been calculated.*, significant intercept; Red, positive intercept; green, negative intercept.

	Response Variable							
	cells per ml				copies per ml			
**Predictor variable**	estimate	SE	statistic	p value	estimate	SE	statistic	p value
Institute A (Intercept)	3.3315	1.2895	2.5836	0.0146*	-1.3511	2.8544	-0.4733	0.6392
Institute B	0.5330	0.8252	0.6459	0.5230	3.9462	1.8266	2.1604	0.0383*
Institute C	3.1072	0.6771	4.5890	0.0001*	7.1063	1.4988	4.7413	4.2E-05*
Institute D	3.1153	0.9079	3.4315	0.0017*	8.8862	2.0096	4.4218	0.0001*
Institute F	3.0519	0.9163	3.3307	0.0022*	7.5415	2.0283	3.7181	0.0008*
Institute G	-0.0865	0.5553	-0.1557	0.8772	-0.2026	1.2293	-0.1648	0.8701
Institute H	2.3258	0.5499	4.2290	0.0002*	3.8412	1.2174	3.1553	0.0035*
Institute I	-4.8709	1.2281	-3.9663	0.0004*	-7.4364	2.7184	-2.7356	0.0101*
Phase: Debris	1.6703	0.5003	3.3387	0.0021*	4.9787	1.1074	4.4957	0.0001*
Storage: LN	1.4425	0.7353	1.9619	0.0585	-0.6000	1.6276	-0.3686	0.7148
Air supply and air exhaust	-5.4767	1.5736	-3.4803	0.0015*	-12.5818	3.4833	-3.6120	0.0010*
number of openings	3.5659	0.9445	3.7752	0.0007*	10.1484	2.0909	4.8537	3.0E-05*
usage time	0.3284	0.1000	3.2831	0.0025*	0.6857	0.2214	3.0968	0.0041*

Table S6. Results overview of the microbial load in liquid nitrogen (LN) storage tanks. The table includes all samples above the determined detection limit in decreasing order of cell counts summarizing important metadata and results. ID, identity number; Deb, Debris; gN, gaseous phase of the LN storage tanks; F, PCR fungi; M, PCR *Mycoplasma*; -, negative; +, positive; [1]*Risk group 1 and 2;* [2]listed according to "Zentrale Kommission für die Biologische Sicherheit*"; [#1]Lactobacillus helveticus; [#2]Leuconostoc mesenteroides, Staphylococcus epidermidis, Staphylococcus equorum, Streptococcus pneumoniae, Streptococcus mitis, Streptococcus thermophilus; [#3]Staphylococcus epidermidis, Streptococcus pneumoniae, Streptococcus mitis, Streptococcus anginosus, Streptococcus agalactiae, Streptococcus sanguinis, Streptococcus mutans, Streptococcus thermophilus; [#4]Acinetobacter calcoaceticus; [#5] Pseudomonas stutzeri; [#6] Acinetobacter oleivorans, Flavobacterium succinicans, Flavobacterium psychrophilum.*

**Institute**	**ID**	**Sampled phase**	**in use (years)**	**Frequency of opening open**	**Storage phase**	**Storage device**	**Stored material**	**Storage condition**	**F**	**M**	**Top 3 Genera**
H	32	Deb	16	daily	mix	mix	mix (bacteria, phages, fungi)	air supply and air exhaust	+	+	*Elizabethkingia*[1,2], *Empedobacter*[1,2], *Janthibacterium*
C	11	ice	4	every second day	gN	tubes	animal (rodent)	air exhaust	+	+	*Pseudomonas*[1,2], *Pedobacter*, *Methylobacterium*[2]
B	4	ice	7	every second day	LN	straws	animal (rodent)	air exhaust	-	-	*Bacteroides*[1,2] *Caldalkalibacillus*, *Psychrobacter*[1]
H	21	ice	16	daily	mix	mix	mix (bacteria, phages, fungi)	air supply and air exhaust	-	-	*Psychrobacter*[1], *Bacteroides*[1,2], *Caldialkalibacillus*
H	25	ice	5	every second day	mix	tubes	mix (human, animal)	air exhaust	-	-	*Bacteroides*[1,2], *Methylobacterium*[2], *Caldialkalibacillus*
C	10	ice	3	every second day	gN	tubes	animal (rodent)	air exhaust	+	-	*Pedobacter*, *Pseudomonas*[1,2], *Methylobacterium*[2]
A	1	ice	9	every second day	gN	bags	human	filtered air supply	-	-	*Bacteroides*[1,2], *Caldalkalibacillus*, *Lactobacillus*[1,2,#1]
B	6	ice	1	every second day	LN	straws	animal (rodent)	air exhaust	-	-	*Bacteroides*[1,2], *Caldalkalibacillus*, *Psychrobacter*[1]
C	9	ice	5	every second week	gN	tubes	animal (rodent)	air exhaust	+	-	*Methylobacterium*[2], *Bacteroides*[1,2], *Caldalkilabacillus*
C	8	ice	6	every second week	gN	tubes	animal (rodent)	air exhaust	-	-	*Streptococcus*[1,2], *Staphylococcus*[1,2], *Leuconostoc*[1,2, #2]
F	17	ice	3	seldom	LN	tubes	plant	filtered air supply and air exhaust	+	+	*Bacteroides*[1,2], *Propionibacterium*[1,2], *Enhydrobacter*
D	12	ice	5	once a month	gN	bags	human	filtered air supply	-	-	*Methylobacterium*[2], *Bacteroides*[1,2],

											Enhydrobacter
A	2	ice	4	daily	gN	bags	human	filtered air supply	-	-	*Paracoccus*[1,2], *Streptococcus*[1,2], *Staphylococcus*[1,2, #3]
A	3	ice	4	daily	gN	bags	human	filtered air supply	-	-	*Streptococcus*[1,2], *Staphylococcus*[1,2], *Paracoccus*[1,2, #3]
F	18	ice	5	seldom	mix	mix	mix (plant, human)	filtered air supply and air exhaust	+	+	*Methylobacterium*[2], *Enhydrobacter*, *Acinetobacter*[1,2, #4]
G	20	ice	10	every second day	gN	tubes	mix (human, animal (rodent, pig, monkey), plant)	hall	-	+	*Flavobacterium*[(2)], *Pseudomonas*[1,2], *Propionibacterium*[1,2, #5]
I	30	ice	16	daily	gN	mix	mix (plant (leaves), animal (fish, mussels, dove egg))	filtered air supply and air exhaust	-	+	*Flavobacterium*[(2)], *Pseudomonas*[1,2], *Acinetobacter*[1,2, #6]
I	29	ice	16	daily	gN	container	mix (plant (leaves), animal (fish, mussels, dove egg))	filtered air supply and air exhaust	+	+	*Methylobacterium*[2], *Acinetobacter*[1,2], *Bacillus*[1,2*]

Table S7. Statistic of α- diversity measures (observed phylotypes, Chao1 and Shannon diversity index) determined in liquid nitrogen (LN) storage tanks after excluding the samples below the determined background . The significance of differences of the α-diversity measures between the different observations of each parameter were calculated (by ANOVA) and shown as compact letter display. A-J, Identification letter of each institute; Deb, Debris; gN, gaseous phase of the LN storage tanks.

Parameter	Observation	Observed	Chao	Shannon
Institute	A	b	c	c
	B	a	b	b
	C	ab	bc	b
	D	a	ab	a
	F	b	c	bc
	G	b	ac	abc
	H	ab	bc	bc
	I	ab	bc	bc
Opening frequency	daily	b	ab	b
	every second day	ab	a	b
	every second week	ab	ab	b
	once a month	a	a	a
	seldom	b	b	b
Storage condition	air exhaust	a	a	a
	air supply and air exhaust	ab	a	a
	filtered air supply	ab	ab	a
	filtered air supply and air exhaust	ab	ab	a
	hall	b	a	a
Stored material	animal	a	a	a
	human	ab	ab	ab
	mix	ab	ab	ab
Storage device	bags	bc	ab	a
	container	a	ab	a
	mix	ac	ab	b
	straws	ab	a	a
	tubes	c	b	a
Storage phase	gN	a	a	a
	LN	a	a	a
	mix	a	a	a
Sampled phase	ice	b	b	b
	Deb	a	a	a
